# SARS-CoV-2 Impairs Dendritic Cells and Regulates DC-SIGN Gene Expression in Tissues

**DOI:** 10.3390/ijms22179228

**Published:** 2021-08-26

**Authors:** Guoshuai Cai, Mulong Du, Yohan Bossé, Helmut Albrecht, Fei Qin, Xizhi Luo, Xiao Michelle Androulakis, Chao Cheng, Mitzi Nagarkatti, Prakash Nagarkatti, David C. Christiani, Michael L. Whitfield, Christopher I. Amos, Feifei Xiao

**Affiliations:** 1 Department of Environmental Health Sciences, Arnold School of Public Health, University of South Carolina, Columbia, SC 29208, USA; 2 Department of Environmental Health, Harvard T.H. Chan School of Public Health, Boston, MA 02115, USA; mulongdu@hsph.harvard.edu (M.D.); dchris@hsph.harvard.edu (D.C.C.); 3 Center for Global Health, Department of Biostatistics, School of Public Health, Nanjing Medical University, Nanjing 211166, China; 4 Department of Molecular Medicine, Institut Universitaire de Cardiologie et de Pneumologie de Québec (IUCPQ), Laval University, Quebec City, QC G1V 4G5, Canada; yohan.bosse@criucpq.ulaval.ca; 5 Prisma Health Medical Group, Department of Internal Medicine, University of South Carolina, Columbia, SC 29208, USA; helmut.albrecht@uscmed.sc.edu; 6 Department of Epidemiology and Biostatistics, Arnold School of Public Health, University of South Carolina, Columbia, SC 29208, USA; fqin@email.sc.edu (F.Q.); xluo@email.sc.edu (X.L.); 7 Neurology, Columbia VA Health System, Columbia, SC 29209, USA; michelle.androulakis@uscmed.sc.edu; 8 Department of Neurology, School of Medicine, University of South Carolina, Columbia, SC 29209, USA; 9 Department of Medicine, Institute for Clinical and Translational Research, Baylor College of Medicine, Houston, TX 77030, USA; Chao.cheng@bcm.edu (C.C.); chris.amos@bcm.edu (C.I.A.); 10 Dan L. Duncan Comprehensive Cancer Center, Department of Medicine, Baylor College of Medicine, Houston, TX 77054, USA; 11 Department of Pathology, Microbiology and Immunology, School of Medicine, University of South Carolina, Columbia, SC 29209, USA; mitzi.nagarkatti@uscmed.sc.edu (M.N.); PRAKASH@mailbox.sc.edu (P.N.); 12 Department of Medicine, Massachusetts General Hospital, Boston, MA 02114, USA; 13 Department of Biomedical Data Science, Geisel School of Medicine at Dartmouth, Lebanon, NH 03756, USA; Michael.L.Whitfield@dartmouth.edu

**Keywords:** COVID-19, dendritic cells, ACE2, DC-SIGN, L-SIGN

## Abstract

The current spreading coronavirus SARS-CoV-2 is highly infectious and pathogenic. In this study, we screened the gene expression of three host receptors (ACE2, DC-SIGN and L-SIGN) of SARS coronaviruses and dendritic cells (DCs) status in bulk and single cell transcriptomic datasets of upper airway, lung or blood of COVID-19 patients and healthy controls. In COVID-19 patients, DC-SIGN gene expression was interestingly decreased in lung DCs but increased in blood DCs. Within DCs, conventional DCs (cDCs) were depleted while plasmacytoid DCs (pDCs) were augmented in the lungs of mild COVID-19. In severe cases, we identified augmented types of immature DCs (CD22^+^ or ANXA1^+^ DCs) with MHCII downregulation. In this study, our observation indicates that DCs in severe cases stimulate innate immune responses but fail to specifically present SARS-CoV-2. It provides insights into the profound modulation of DC function in severe COVID-19.

## 1. Introduction

In 2020, a novel coronavirus SARS-CoV-2 has been spreading as a pandemic infection. SARS-CoV-2 is highly infectious and pathogenic through human-to-human transmission and causes severe Coronavirus Disease 2019 (COVID-19) [1]. COVID-19 critical cases are often characterized by a pro-inflammatory “cytokine storm” with a release of excessive cytokines (including IL-6, IL-1, IL-2, IL-10, TNF-α and IFN-γ and others) and a hyperactive immune response which causes damage to target organs. Despite extensive research in the past year, the exact immune pathophysiology of SARS-CoV-2 has remained elusive.

Studies have showed that SARS-CoV-2 is closely related to SARS-CoV, with around 80% homology of genome [2]. They belong to the same β-genus of coronavirus with similar receptor-binding domain (RBD) structures [3] and cause similar clinical symptoms such as acute respiratory response [4]. Many viruses use alternative receptors to enter host cells. For SARS-CoV, three receptors including ACE2, DC-SIGN and L-SIGN (gene symbols *ACE2*, *CD209* and *CLEC4M*, respectively) have been found to be involved in the pathogenicity [5,6,7], among which ACE2 has been quickly confirmed to be the receptor for SARS-CoV-2 [2]. DC-SIGN and L-SIGN have also been considered as potential receptors for SARS-CoV-2 [8,9]. However, the associations between DC-SIGN/L-SIGN expression and SARS-CoV-2 infection and its phenotypes were not evaluated yet.

In vivo, L-SIGN is largely expressed on endothelial cells in liver sinusoids and lymph nodes, whereas DC-SIGN is mainly expressed on dendritic cells (DCs) [10]. DC-SIGN and L-SIGN provide gateways for SARS coronavirus to attack immune cells. Indeed, aside from epithelial cells, SARS-CoV infection was also found in T cells, macrophages (Mø) and monocyte-derived dendritic cells and this viral attack is probably responsible for the lymphopenia which was commonly observed in SARS-CoV infected patients [11]. On the other hand, DC-SIGN and L-SIGN play important anti-viral roles that they both capture, transmit and disseminate virus within the host [12,13]. With the dual roles of DC-SIGN as a potential SARS-CoV-2 gateway and an innate immune initiator, DCs may play important and complicated roles in SARS-CoV-2 infection and clinical outcomes of COVID-19 patients. Studies have indicated a decrease of conventional DCs (cDCs) and plasmacytoid DCs (pDCs) in the lung and blood of severe COVID-19 cases [14,15]. Yet, the modulation of DCs and its DC-SIGN gene expression across airway and peripheral blood tissues with COVID-19 infection have not been characterized.

**Figure 1:**
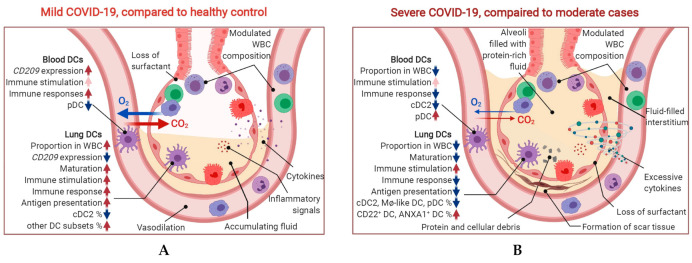
Graphical abstract. The findings from this study in lung and blood DCs were summarized for mild cases vs. healthy control (**A**) and severe cases vs. mild cases (**B**). Highlighted points are (1) SARS-CoV-2 modulates the DCs proportion and *CD209* expression differently in lung and blood; (2) severe infection is characterized by DCs less capable of maturation, antigen presentation and MHCII expression; (3) the proportion of cDCs was decreased while that of pDC was increased in lung with SARS-CoV-2 infection but immature subsets (CD22^+^/ANXA1^+^) were accumulated in severe cases.

To investigate the variation of DC status and DC-SIGN expression in COVID-19 cases, in this study, we systematically studied the transcriptome in single cells in the upper airway (nasopharynx/pharynx samples), lower airway (bronchoalveolar lavage fluid (BALF) samples) and peripheral blood (peripheral blood mononuclear cell (PMBC) samples) of COVID-19 patients with different severity levels. The findings of this study were illustrated in Figure 1.

Compared to healthy controls, the expression of DC-SIGN was found to be increased in blood while decreased in lung of COVID-19 patients. In severe cases, we found significantly decreased DCs and its augmented immature subsets, which was not observed in mild patients. These results illustrate the pathogenicity and virulence of SARS-CoV-2 infection from the aspect of DCs which is a critical player in the immune response to viral infection.

## 2. Results

### 2.1. DC-SIGN Is Associated with SARS-CoV-2 Infection and Immune Cell Activation

**Figure 2:**
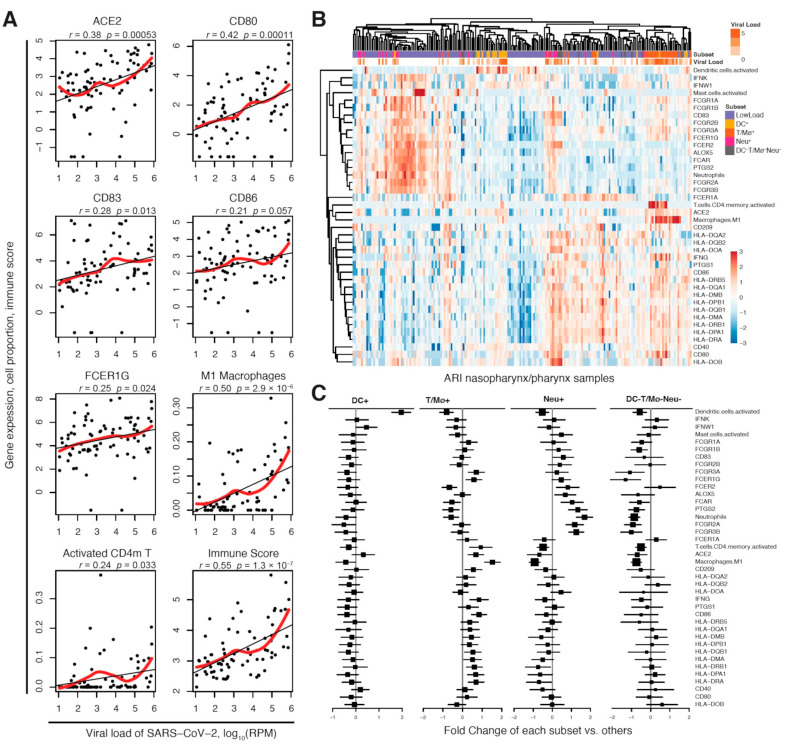
Correlation and variation of SARS-CoV-2 receptors and immune factors in nasopharynx/pharynx samples of SARS-CoV-2 ARIs. (**A**) Scatter plots of SARS-Cov-2 viral load against gene expression profiles of SARS-CoV-2 receptor ACE2, immune response activators (CD80, CD83 and CD86), FCER1G, the proportion of active CD4 memory T cells and M1 macrophages in 22 immune cell types, as well as scores from a linear model of 5 immune cell types (active CD4 memory T cells, M1 Mø, mast cells, neutrophils and active DCs). The black and red lines are fits of linear model and LOESS, respectively. (**B**) Hierarchical clustering and heatmap of SARS-CoV-2 receptors, immune factors and proportions of 5 immune cell types in ARI samples. Five subsets of ARI samples were identified, including 1 subset with low SARS-CoV-2 load (LowLoad) and 4 subsets with high SARS-CoV-2 load and activated DCs (DC^+^), activated T cells or M1 Mø (T/Mø^+^), activated neutrophiles (Neu^+^) and no activation of any of those cell types (DC^−^T/Mø^−^Neu^−^). For each feature, red indicates relatively higher value and blue indicates relatively lower value across samples. The scale corresponds to the relative value across samples. (**C**) The fold changes of each feature comparing each subset to others. 95% confidence intervals are shown as bars. The size of the squares is proportional to the standard deviation.

In the metagenomic next generation sequencing (mNGS) data from nasopharynx/pharynx samples of 197 patients with acute respiratory illnesses (ARIs), we found gene expression of ACE2 ($p=3.9\times 10^{-10}$) and DC-SIGN (p=0.048) were significantly higher with the SARS-CoV-2 infection (Supplementary Figure S1). No data was available for *CLEC4M*. Notably, the gene expression of essential factors of antigen-presenting cells (APC) for antigen presentation including CD40, CD80 and CD86, HLA genes, IgG immunoglobulin receptor (FcR) III and interferon-gamma (IFN-γ) were upregulated in SARS-CoV-2 ARIs compared to non-viral ARIs, whereas gene expression of IgA and IgE FcRs (FCER1A, FCER2), interferon-kappa (IFN-κ), and cyclooxygenase (COX) (PTGS1 and ALOX5) were downregulated. In SARS-CoV-2 ARIs, the gene expression of ACE2, CD80, CD86, CD83, IFN-γ, FCER1G and IgG FcRs were positively associated with the viral load of SARS-CoV-2 (Figure 2A, Supplementary Figure S1), while no significant associations were observed in HLA genes. In addition, we infer the composition of 22 immune cells (see Methods: Upper airway samples from COVID-19 patients and controls) in samples and found the proportions of activated CD4^+^ memory T cells (CD4m T) and M1 Mø were increased in SARS-CoV-2 ARIs compared to non-viral ARIs and significantly associated with increased viral load (Figure 2A, Supplementary Figure S1), while the proportions of activated mast cells and neutrophils were decreased in SARS2-CoV-2 ARIs (Supplementary Figure S1). The proportions of these four types of immune cells and the active DCs well predicted the SARS-CoV-2 load ($r=0.55,\;p=1.3\times 10^{-7}$, Figure 2A) and discriminated SARS-CoV-2 ARIs from non-viral and ARIs ($p=1.2\times 10^{-5}$, Supplementary Figure S1).

Interestingly in ARIs with relatively high SARS-CoV-2 viral load (log_10_ CPM > 1), we observed distinct subtypes associated with the activation of DCs and T/Mø cells, with specific immune cell compositions and specific expression of APC antigen reorientation factors and SARS-CoV-2 receptors (Figure 2B). According to the proportion of the five associated immune cell types (i.e., DCs, CD4m T, M1 Mø, active mast cells and neutrophils), we therefore further classified ARIs into four subtypes, including the DCs-enriched subtype (DC^+^) and the T cells or Mø-enriched subtype (T/Mø^+^), the neutrophils-enriched subtype (Neu^+^) and the triple negative subtype which was without enrichment of any of these cells (DC^−^T/Mø^−^Neu^−^) (Figure 2B). The DC^+^ subtype was characterized with activated DCs, downregulated FcRs and HLA genes; the T/Mø^+^ subtype was characterized with activated T or Mø, upregulated viral gateway *ACE2* and *CD209* as well as immune factors including *INFG*, *CD86*, *FCGR3A*, *FCGR1G* and HLA genes; the Neu^+^ subtype was characterized with enriched neutrophils, upregulated FcRs genes and Cox genes (*PTGS2* and *ALOX5*), and downregulated HLA genes and *ACE2*; the DC^−^T/Mø^−^Neu^−^ subtype was characterized with downregulated FcRs genes, *ACE2* and *CD209*. These results provide new clues for the variation of immune response in COVID-19 patients by its potential link to the differential activation of specific immune cell types. Not like the T/Mø^+^ subtype with activated immune factors and high expression of *ACE2* and *CD209*, DC^+^, Neu^+^ and DC^−^T/Mø^−^Neu^−^ subtypes may produce the high virus load by immunodeficiency.

### 2.2. DC-SIGN Is Associated with COVID-19 Severity, Immune- and Neural-Related Phenotypes and Respiratory Diseases

We investigated the association of the expression of ACE2, DC-SIGN and L-SIGN with COVID-19 risk by large-scale (n=459,250) population genetic association analyses (see Methods: Mendelian Randomization analysis). Using Mendelian Randomization (MR) with the inverse variance weighted method, we found that higher expression of DC-SIGN plasma protein was associated with increased COVID-19 risk ($\beta =0.14,\;p=3.39\times 10^{-3}$) and severity ($\beta =0.30,\;p=5.41\times 10^{-5}$; Supplementary Table S1). No significant associations were found in ACE2 or L-SIGN.

The important role of DC-SIGN in phagocytosis and immune activation was also reflected in associations with phenotypes. Genome wide association study (GWAS) (see Methods: Transcriptome-based phenome-wide association study) found that the genetic variants of *CD209* were associated with multiple diseases, including asthma (the top among detected associations, $p=1.0\times 10^{-13}$), cancers and neurological disorders (Supplementary Table S2), showing their possible underlying links involving DCs and/or Mø (which also express CD209 on surface).

We furthermore investigated the influence of *CD209* gene expression on the immune cell composition in the lung tissue. The transcriptome-based PheWAS analysis found the significant association of *CD209* gene expression with the depletion of neutrophils (count: $\beta =-0.10,\;p=4.0\times 10^{-5}$; percentage: $\beta =-0.60,\;p=3.2\times 10^{-5}$) and overall white blood cells (WBC) (count: $z=-3.34,\;p=8.3\times 10^{-4}$), as well as the augmentation of lymphocytes (percentage: $\beta =0.14,\;p=3.00\times 10^{-4}$) and monocytes (percentage: $\beta =0.15,\;P=1.24\times 10^{-3}$) (Supplementary Table S3). These associations were further confirmed by MR analysis on DC-SIGN plasma protein expression (neutrophil count: $\beta =-0.03,\;p=1.38\times 10^{-16}$; neutrophil percentage: β=−0.02, p=0.02; WBC count: $\beta =-0.03,\;p=5.40\times 10^{-27}$; lymphocyte percentage: $\beta =0.02,\;p=5.29\times 10^{-5}$; monocyte percentage: $\beta =0.03,\;p=8.52\times 10^{-3}$; Supplementary Table S4). Moreover, we also observed such significant associations of *CD209* expression with immune cell composition in blood samples of 160 Acute Respiratory Distress Syndrome (ARDS) cases and 142 non-ARDS controls, regardless of the disease status (Supplementary Figure S2, see Methods: Blood samples from ARDS cases and controls). In addition, we found that *CD209* expression in lung was associated with immune related symptoms such as tiredness or low energy, depression and duration of fitness (Supplementary Table S3). In lung adenocarcinoma tumors from The Cancer Genome Atlas (TCGA, https://www.cancer.gov/tcga, accessed on 19 January 2020), we only observed a slight downregulation of *CD209* (β=−0.26, p=0.07) compared to normal tissues. No significant association was observed with survival (p=0.33) or tumor stage (p=0.80).

### 2.3. CD209 Expression in DCs and Mø Is Associated with COVID-19 Severity

**Figure 3:**
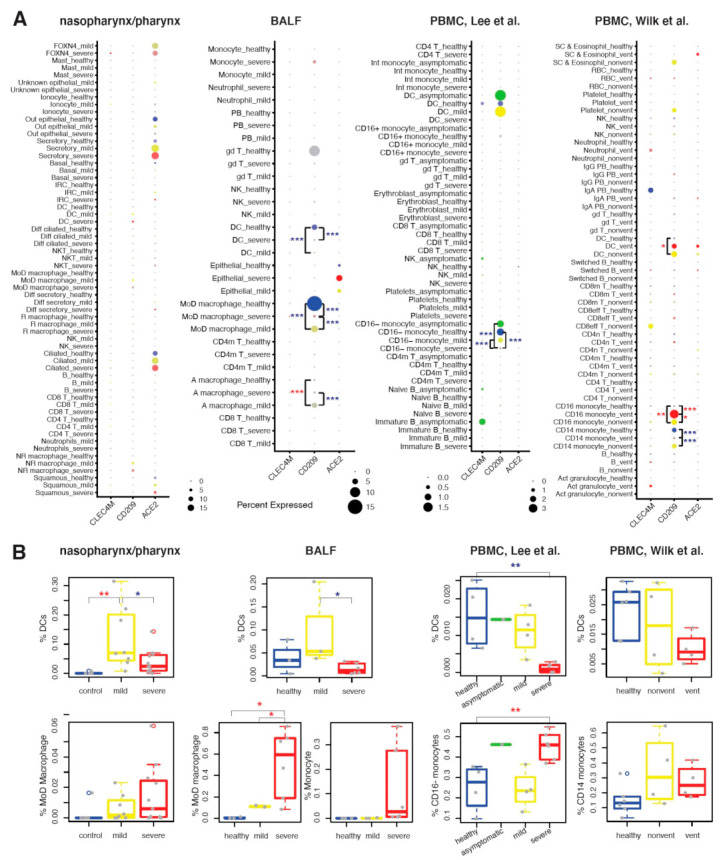
Gene expression differences of three SARS-Cov-2 receptors in upper airway, lung and blood of healthy and COVID-19 cases. (**A**) Variations of expression profiles of *ACE2*, *CD209* and *CLEC4M* in cell types identified from four scRNA-seq datasets of nasopharynx/pharynx, BALF and PBMC samples from COVID-19 patients and healthy controls. For each dataset, the detection rate and the average expression level of each receptor in identified cell types are illustrated. The detection rate was showed by dot size and a larger size indicates a higher detection rate. The average expression level is showed by the color scale of dot, from grey to blue (for healthy control), green (for asymptomatic cases), yellow (for mild cases) and red (for severe cases). A darker color indicates a higher expression level. The gd T cell in healthy BALF samples was shown in grey because only a limited number of cells was detected (n=20). (**B**) Variations of the proportions of DCs, macrophages or monocytes in WBC in samples from COVID-19 patients and healthy controls. For each group of disease status, cell proportions were shown in boxes and data from a particular participant was shown by a dot. In DCs, monocytes and monocytes, differences in (**A**) gene expression and (**B**) cell proportions were tested in severe cases compared to mild cases, in severe cases compared to healthy controls, in mild cases compared to healthy controls, in severe cases compared to asymptomatic cases, in mild cases compared to asymptomatic cases, and in asymptomatic cases compared to healthy controls. Significance was shown by red * for higher mean value and blue * for lower mean value, with * *p* < 0.05; ** *p* < 0.01; *** *p* < 0.001.

To investigate the association of CD209 expression and DCs with COVID-19 risk, we next systematically investigated the cell type-specific expression of *CD209* in upper airway, lower airway and peripheral blood. scRNA-seq datasets from one nasopharynx/pharynx, one BALF and two PBMC (Lee et al. [16], Wilk et al. [15]) studies from COVID-19 patients and healthy controls were analyzed. With identified cell types in each dataset (Supplementary Figure S3), the detection rates and average expression of *ACE2*, *CD209* and *CLEC4M* were compared between healthy controls, mild cases and severe cases (Figure 3A). Compared to healthy controls, patients with mild and severe COVID-19 showed higher detection rates of *ACE2* in the nasopharynx/pharynx secretory, ciliated, and pulmonary epithelial cells. No notable expression of *CLEC4M* was observed in any cell type in any of the four datasets.

Notable expression of *CD209* was found in DCs, airway Mø and blood monocytes (Figure 3A). Significantly, we found that *CD209* expression in lung monocyte-derived macrophages (MoD Mø) and blood classical (CD16^−^ or CD14^+^) monocytes was severity-dependently decreased in samples from COVID-19 patients, while blood CD16^+^ monocytes showed a severity-dependent increase of *CD209* expression in the dataset of Wilk et al. [15]. In DCs, we found *CD209* expression was decreased in lungs but increased in blood of COVID-19 cases, as compared to healthy controls. With a small number of detected DCs (N = 12), the Lee PBMC dataset did not detect such increase in blood of severe COVID-19 cases. The discrepancy among anatomical sites were also found in the alteration of the DC proportion within WBC (Figure 3B). In airway samples, the proportion of DCs was increased in mild cases and decreased in severe cases. A decrease was also found in severe cases in blood, but no notable change was found in mild cases. Inversely to DCs, the proportions of MoD Mø and monocytes were increased in severe cases in all three tissues. These may indicate a site-specific impact of COVID-19 on the differentiation of monocytes to DCs and Mø, and further studies are warranted.

### 2.4. Maturation and Antigen Presentation Ability of DCs Are Inhibited in Both Lung and Blood of Severe COVID-19

**Figure 4:**
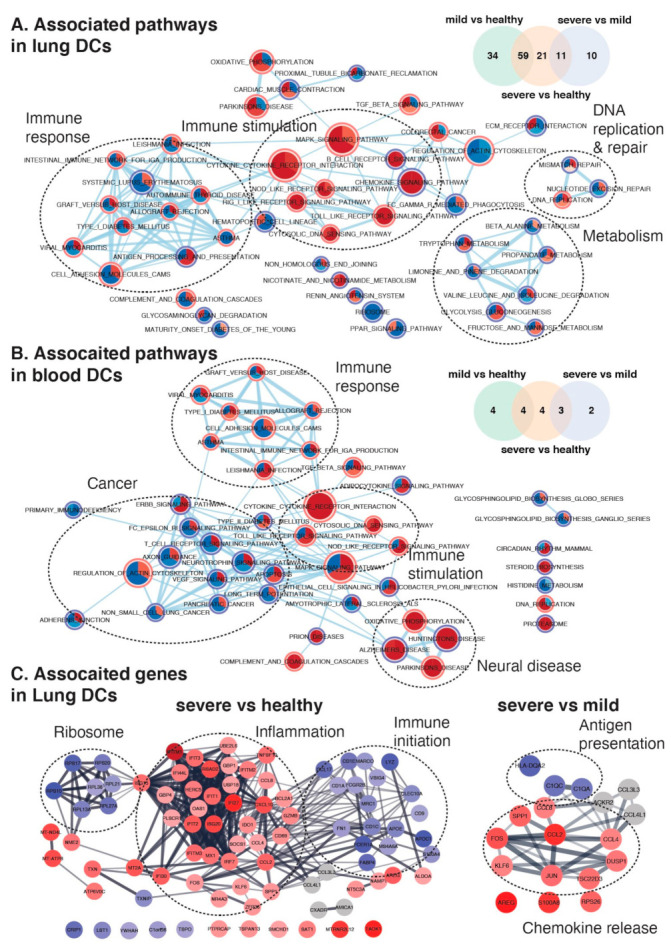
Dysregulated gene expression networks in lung and blood with COVID-19. (**A**,**B**) show pathways associated with SARS-CoV-2 infection and disease severity in lung and blood DCs separately. Each node in network is a pie plot showing three comparisons, including mild vs healthy (top right), severe vs healthy (bottom), and severe vs mild (top left). Node size corresponds to the number of genes in dataset within the pathway. Color intensity inner the node corresponds to the significance of the pathway for this dataset (red is for positive effect; blue is for negative effect, darker indicates larger effects). Orange outer circles show the overlapping pathways found in lung and blood DCs, while blue outer circles show the uniquely detected pathways. Edge weight corresponds to the number of genes found in both connected pathways. Venn diagrams show the distribution of genes with more than 2 folds alterations which were detected from above three comparisons in each dataset. (**C**) shows STRING networks of changed genes in lung DCs in severe COVID-19 cases compared to healthy (left) and mild cases (right). The intensity of filling color corresponds to the log_2_ fold-of-change of the gene expression (red is for positive effect; blue is for negative effect).

We further studied transcriptome-wide differently expressed genes (DEGs) and signaling pathways in DCs in COVID-19 lungs and blood (Figure 4). Compared to healthy controls, an extensive transcriptomic change was found in lung tissue that 184 DEGs (93 for mild; 91 for severe, Figure 4A) showed expression changed more than 2-fold. Differently, such alterations were detected on only 15 genes (eight for mild; 11 for severe, Figure 4B) in blood. Relatedly, we also found a broad COVID-19 severity-associated activation of viral detection and immune stimulation (cytokine-cytokine receptor interaction, cytosolic DNA sensing, TLR, Nod-like receptor (NLR), chemokine signaling, B cell receptor signaling and Mark signaling) in lung, whereas only mild immune stimulation (cytokine-cytokine receptor interaction and cytosolic DNA sensing) was found in blood. In both lung and blood, immune response related pathways (graft versus host disease, viral myocarditis, Leishmania infection and others) were activated in mild cases as compared to healthy control but interestingly were inhibited in severe cases as compared to mild cases. Such pattern was also observed on antigen processing and presentation, host metabolism as well as DNA replication in lung but not in blood. Correspondingly, immune initiation related genes (including *CD1A*, *CD1C*, *CD1E*, *CCL17*, *FCER1A*, *FCGR2B* and others) and ribosome genes were significantly downregulated in the lungs of severe COVID-19 cases as compared to healthy controls, whereas inflammatory signals (including interferon genes, *CCL2*, *CCL4*, *CCL8* and others) were upregulated (Figure 4C). Compared to mild cases, chemokines (including *CCL2*, *CCL4*, *CCL8*) and MAPK substrates (including *JUN*, *USF1*, *FOS*) were upregulated in the lungs of severe cases, whereas antigen presentation related genes (*HLA-DQA2*, *C1QC* and *C1QA*) were downregulated. Interestingly in blood, we also found neural disease related pathways were severity-dependently activated and cancer related pathways were inhibited in severe cases (Figure 4B), which may link to the neurologic and other clinical manifestations of COVID-19.

**Figure 5:**
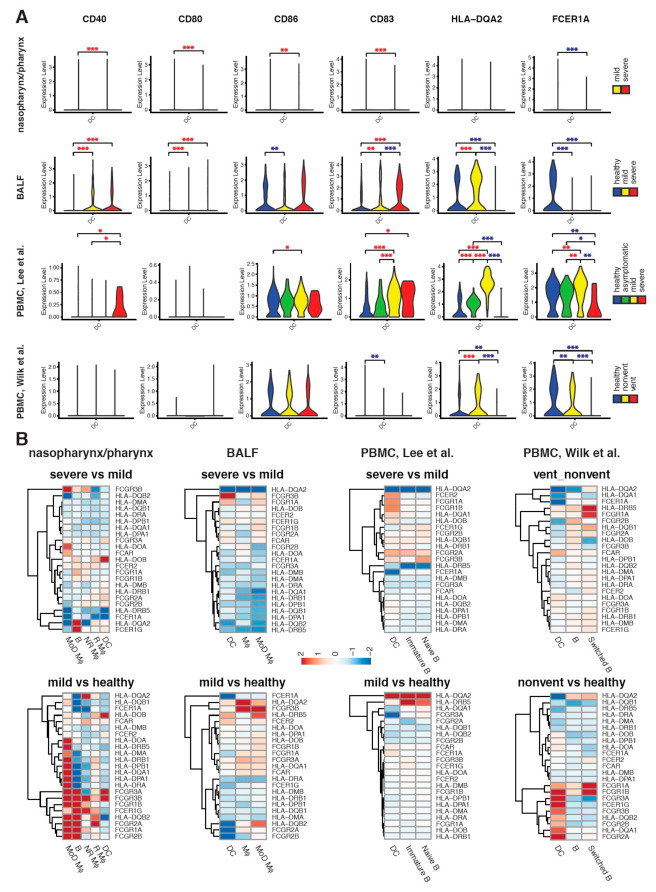
The expression profiles of functional markers in DCs at three sites. (**A**) The expression disparity of markers of maturation (CD83), T-cell stimulation molecules (CD40, CD80, CD86) and antigen presentation molecules (HLA-DQA2 and FCER1A) in DCs of COVID-19 cases and healthy controls. One upper airway (nasopharynx/pharynx), one lung (BALF) and two blood (PBMC) scRNA-seq datasets were analyzed. The expression of each gene is shown in violin plots. For some of genes which has low detection rate, the violin plot displays a bar. Differential expression was tested in severe cases compared to mild cases, in severe cases compared to healthy controls, in mild cases compared to healthy controls, in severe cases compared to asymptomatic cases, in mild cases compared to asymptomatic cases, and in asymptomatic cases compared to healthy controls. Significance was shown by red * for higher mean value and blue * for lower mean value, with * *p* < 0.05; ** *p* < 0.01; *** *p* < 0.001. Despite interest, we did not compare COVID-19 nasopharynx/pharynx samples to healthy controls due to the limited detection of DCs in healthy samples (N = 1). (**B**) The dysregulation of gene expression of MHCII and FcRs in APCs at three sites with COVID-19. For each gene, the alteration of gene expression in APCs of severe COVID-19 cases compared to mild cases, and that in mild COVID-19 cases compared to healthy controls are visualized in heatmaps. Red indicates upregulation and blue indicates downregulation. The scale corresponds to log_2_ fold-of-change.

The dysregulation of DC function in COVID-19 was also presented by the expression of the maturation marker (*CD83*), T-cell stimulation molecules (*CD40*, *CD80*, *CD86*) and antigen presentation molecules (*HLA-DQA2* and *FCER1A*) (Figure 5A). In lung, *CD40*, *CD80*, *CD83* and *HLA-DQA2* were upregulated while *CD86* and *FCER1A* were decreased in mild COVID-19 as compared to healthy controls. Similar patterns were observed on *CD40*, *CD86* and *HLA-DQA2* in both blood datasets while inconsistent results of *CD83* and *FCER1A* were observed in Lee and Wilk PBMC datasets. Compared to mild cases, severe COVID-19 showed upregulated *CD40* together with downregulated *CD83*, *HLA-DQA2* and *FCER1A* in all three tissues. Interestingly, asymptomatic cases showed similar expression of those markers with healthy controls in blood.

Further, we investigated the expression of MHC class II molecules (MHCII) and Fc receptors (FcRs) and found that they were broadly upregulated in APCs in lungs of mild COVID-19 patients, with a MHCII subset (*HLA-DRA*, *HLA-DMB*, *HLA-DRB1*, *HLA-DPB1*, *HLA-DQB1*, *HLA-DMA*) were downregulated (Figure 5B). Compared to mild cases, severe cases showed decreased MHCII expression in airway APCs but not significantly in blood. The result was in agreement with above pathway analysis that antigen processing and presentation were activated in mild cases but inhibited specifically in lungs of severe cases. In both lung and blood, HLA-DQA2 expression in APCs was significantly increased in mild cases as compared to healthy controls but downregulated in severe cases as compared to mild cases. Interestingly, compared to healthy controls, the global change of MHCII and FcRs gene expression in blood were associated with COVID-19 severity, with upregulation in asymptomatic cases, no significant change in mild cases and downregulation in severe cases (Supplementary Figure S4).

Together, these observations indicate the ability of DCs for T-cell stimulation is induced but the maturation of DCs is inhibited in both lung and blood of severe COVID-19, and the antigen presentation ability of APCs is significantly reduced in lung, which is the primary site of infection.

### 2.5. Conventional DC Subsets Were Depleted and New Immature DC Subsets Were Accumulated in Both Lung and Blood of Severe COVID-19

**Figure 6:**
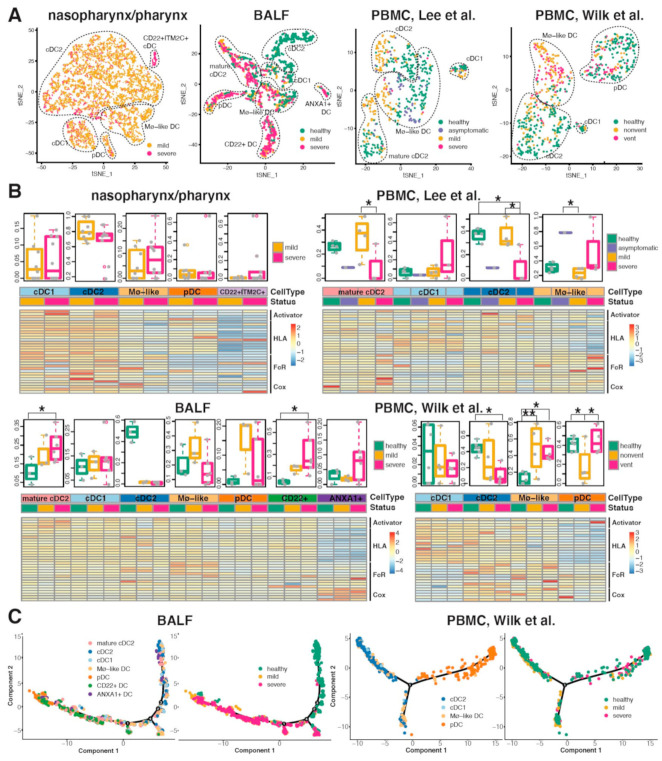
The variation of distributions and transcriptome profiles of DC subsets in COVID-19 samples and healthy controls at three sites. One upper airway (nasopharynx/pharynx), one lung (BALF) and two blood (PBMC) scRNA-seq datasets were analyzed. (**A**) shows DC subsets identified in single-cell transcriptome profiles from COVID-19 mild and severe cases and healthy controls. (**B**) shows the proportions (top) and expression of key players (bottom) of DC subsets in samples from each participant. The differences of proportion of each DC subset were tested in groups. Significance: * *p* < 0.05; ** *p* < 0.01. The average gene expression of the T-cell stimulation activators (CD83, CD40, CD80, CD86), MHCII and FcRs in subsets in samples with different disease status were shown in the bottom heatmap. Red indicates relative higher expression and blue indicates relative lower expression. The scale corresponds to the log_2_ (normalized scRNA-seq read count) which was centered and scaled by row. (**C**) Single-cell trajectory of DCs inferred by Monocle from scRNA-seq data of BALF and PBMC samples. For each dataset, the left shows ordered cells labeled with cell types, while the right shows ordered cells from COVID-19 mild and severe cases, and healthy controls.

We further investigated the variation of DC subsets in nasopharynx/pharynx, BALF and PBMC samples from COVID-19 patients and healthy controls. Based on known markers (Supplementary Figure S5), four DC subsets were identified in the nasopharynx/pharynx samples, including type I and II conventional dendritic cells (cDC1 and cDC2), Mø-like DC which expressed Mø markers S100A8 and S100A9, Plasmacytoid dendritic cell (pDC) and a new subset of CD22^+^ITM2C^+^ DC; seven subsets were identified in the BALF samples, including cDC1, cDC2, mature cDC2, Mø-like DC, pDC and new subsets CD22^+^ DC and ANXA1^+^ DC; four subsets were identified in the PBMC samples from the Lee dataset, including cDC1, cDC2, mature cDC2 and Mø-like DC; four subsets were identified in the PBMC samples from the Wilk dataset, including cDC1, cDC2, mature cDC2 and Mø-like DC (Figure 6A). The integration of all datasets found that these subsets were conservative among tissues (Supplementary Figure S6). CD22^+^ITM2C^+^ DC in nasopharynx/pharynx samples were clustered together with CD22^+^ DC in BALF samples, which were characterized by the activated immune response ($q=1.17\times 10^{-5}$) and interferon gamma response ($q=1.17\times 10^{-5}$) based on KEGG pathway enrichment analysis [17].

Among the whole population of DCs, the proportion of cDC2 was severity-dependently decreased in both lung and blood of COVID-19 (Figure 6B). Inversely, the mature cDC2 was increasingly correlated with disease severity in lung BALF samples. Such increase was also found in PBMC with mild COVID-19 but not in severe cases which might be due to its limited cell detection of DC cells (N = 12). pDC showed a notable augmentation in BALF and depletion in PBMC in mild cases but not in severe cases, which may reflect the different migration activeness of pDC from blood to lung in mild and severe COVID-19. Interestingly, all tissues in severe cases showed augmentation of immature DC subsets with relatively inactively expressed mature markers *CD83*, T-cell stimulation activators (*CD40*, *CD80*, *CD86*) and HLA genes, including CD22^+^ITM2C^+^ DC and Mø-like DC in nasopharynx/pharynx samples, ANXA1^+^ DC and CD22^+^ DC in BALF samples, Mø-like DC in PBMC samples. This may explain the immunosuppression in severe patients of COVID-19. Indeed, in nasopharynx/pharynx samples, the proportion of CD22^+^ITM2C^+^ DC was significantly positively correlated with that of inactive immune cells including M0 Mø ($p=8.6\times 10^{-5}$), resting CD4 memory T cells (p=0.028) and resting mast cells (p=0.024) (Supplementary Figure S7). In addition, we found the BALF specific ANXA1^+^ DC subset showed high expression of COX related genes *PTGS1*, *PTGS2*, *HPGDS*, *ALOX5* and *LTC4S*, which may be responsible for the DC immunity suppression [18], together with *ANXA1* [19].

We observed a clear shift of the proportion of DC subsets of conventional DCs and pDC on single cell trajectory in COVID-19 BALF and PBMC, in response to viral infection (Figure 6C and Supplementary Figure S8). Interestingly, such shift in BALF samples was not observed in severe cases but was “trapped” in immature subsets (CD22^+^ and ANXA^+^ DCs) between conventional DCs and pDC on trajectory. The “blockade” was also observed in nasopharynx/pharynx with severe COVID-19 (Supplementary Figure S9). Further we found that both transcriptional bursting frequency and size of MCHII genes were inhibited in the lung specific immature subset ANXA1^+^ DC as compared to cDC2 and pDC (Supplementary Figure S10). Increased frequency and size of Cox genes were also observed. These indicate that the SARS-CoV-2 infection may inhibit the DC maturation by regulating the expression kinetics parameters (i.e., bursting frequency and bursting size) of related genes.

## 3. Discussion

ACE2, DC-SIGN and L-SIGN are three potential receptors of both SARS-CoV and SARS-CoV-2. No available data indicate they are dependent viral entry portals, and we did not find significant correlations of the gene expression between ACE2 and that of DC-SIGN and L-SIGN (Supplementary Figure S11). Given that DC-SIGN is mainly expressed on the surface of DCs and Mø, the viral infection to immune cells may directly link to the immunopathogenesis and disease severity of COVD-19. Indeed, we found higher gene expression of *ACE2* and *CD209* in upper airway cells of COVID-19 ARIs compared to non-viral ARIs, and *CD209* plasma protein expression was correlated COVID-19 risk and severity. We also observed their association with the variation of immune cell activation in upper airway cells of COVID-19 ARIs that high expression of *ACE2* and *CD209* was only found in the T/Mø activated cells. This might be a result from the immune stimulation by intensive viral attack to ACE2 and DC-SIGN expressing cells. This may also reflect the positive correlation of *CD209* with T cell percentage and negative correlation with neutrophils, which were detected by our large-scale population genetic association study. Indeed, *CD209* mediates the activation of T cells [20]; the activated neutrophils produce TNF-alpha and induce DC maturation which lower the *CD209* expression [21]. The neutrophil-to-lymphocyte ratio has been studied as a severity marker in COVID-19 [22] and as a valid prognostic factor in various solid tumors and other chronic diseases [23]. Therefore, the interaction of *CD209* and DCs with neutrophils and T cells may play an important role in mediating cytokine storm coupled with limited anti-viral capability, which are keys in COVID-19 pathogenesis [24]. Further investigation is required to elucidate this interaction and the causal effect of *ACE2* or *CD209* to the COVID-19 risk.

For the first time, we performed a systematic investigation of the expression of SARS-CoV-2 receptors in single cells of upper airway, lower airway and blood of COVID-19 patients. We found *CD209* expression and the percentage of DCs and MoD Mø or monocytes were associated with COVID-19 severity. Interestingly, the associations were site specific. In the lung and upper airway with mild infection, DCs were induced and augmented for an active immune response. In the cases of severe infection, the decrease of DCs proportion and maturation could be a sign of the exhaustion of DCs. Also, the decreased expression of *CD209* in DCs of COVID-19 lungs may be a result of cell death caused by direct viral infection to DC-SIGN expressed DCs. Differently, blood showed induced DC activation and increased *CD209* expression, which may indicate that DCs are stimulated in blood but not primarily attacked by virus. Indeed, no SARS-CoV-2 sequencing reads were detected from COVID-19 PMBC [15]. Interestingly, DCs in lungs of severe COVID-19 showed upregulation of genes for T cell stimulation but downregulation of genes for antigen presentation and maturation, indicating that DCs may stimulate innate immune system but fail to present SARS-CoV-2. This may lead to the cytokine storm but the failure of anti-viral response in severe COVID-19 patients. SARS-CoV-2 might use an escape mechanism similar to HIV-1 which hides in DCs and uses DC-SIGN as a trans-receptor for efficient transmission to T cells, and also similar to *Mycobacteria tuberculosis* (*M. tb*) which subverts DCs by targeting DC-SIGN and inhibiting maturation [25]. The stimulated and “blinded” immune systems could be responsible for the cytokine storm as well as severe ARDS suffering COVID-19 critical cases. MHCII genes especially HLA-DQA2 might be involved in this pathogenesis, which were found to be significantly decreased in lung of severe cases compared to mild cases. This may also link to the olfactory impairment which is often seen in patients with COVID-19, given that MHC are essential in individual olfactory perception [26].

A broad downregulation of MHCII genes in APCs was observed and aligned with findings that dysfunctional monocytes [27,28] and B cells [29] in severe COVID-19. Here, we report a shift of the proportion of cDCs and pDCs within DCs in COVID-19 cases, which leads to the marked disease severity-dependent depletion of cDC2 in all studied tissues. Strikingly, such shift was not found in severe cases, in which immature subsets including CD22^+^ DC and ANXA1^+^ DC with suppressed activation markers were accumulated. This may cause the immunosuppression of DCs and contribute to the variation of disease severity through the interactions between DCs and T cells and other immune cells. Indeed, we found the immature CD22^+^ITM2C^+^ DCs was significantly positively correlated with the resting Mø, CD4mT and mast cells in upper airway. Further studies are needed to elucidate if the accumulation of immature subsets in severe cases directly blocks the activation of mature DCs and pDC.

Collectively, this study provides important pieces of puzzle of COVID-19 about dysfunctional DC cells from the expression of receptors of SARS coronavirus, remodeling of DC activation and redistribution of DC subsets. Although further studies with a larger sample size of single cell or single cell type studies will be needed to properly assess this matter, this study provide important hints for vaccine development, as well as the variation of COVID-19 severity and pathogenesis.

## 4. Materials and Methods

### 4.1. Bulk Transcriptomics

#### 4.1.1. Upper Airway Samples from COVID-19 Patients and Controls

We studied the metagenomic next generation sequencing (mNGS) data of nasopharynx/pharynx samples from 197 patients with acute respiratory illnesses, including 94 patients confirmed with SARS-CoV-2 infection by clinical PCR and 103 with no virus detected [30]. The host gene expression read counts and the CPM (read counts for million) of SARS-CoV-2 virus were analyzed. We normalized the read counts of *ACE2* and *CD209* using the TMM method and calculated CPM using edgeR [31]. The data for *CLEC4M* was not available.

We deconvoluted the immune cell composition from these bulk RNA-seq data using CIBERSORT [32] with its signature matrix, LM22. In each sample, the proportions of 22 human hematopoietic cell types were inferred, including naïve and memory B cells, plasma cells, seven T cell types (CD8, CD4 native, memory resting CD4, memory activated CD4, follicular helper, regulatory (Tregs) and gamma delta), NK cells (resting and activated), monocytes, macrophages (M0, M1 and M2), dendritic cells (resting and activated), mast cells (resting and activated), eosinophils and neutrophils. Using transcriptome profiles of DC subsets identified in below scRNA-seq datasets of nasopharynx/pharynx samples, we also estimated the composition of DC subsets in these bulk RNA-seq data using MuSiC [33].

Linear regression was used to test the difference of gene expression or immune cell proportions between sample groups as well as their association with SARS-CoV-2 viral load in samples.

Within samples of the 94 COVID-19 patients, we further identified one subtype of low SARS-CoV-2 load (LowLoad) with SARS-CoV-2 viral load log10 CPM ≤ 1, as well as four subtypes based on the proportion of these associated immune cells. Specific cutoffs of cell proportions were applied for each immune cell type, that 0.1 is for DCs, 0.15 for neutrophils, 0.05 for T cells and macrophages. As shown in Supplementary Figure S12, samples that were relatively enriched by each type of immune cells were identified based on these cutoffs.

#### 4.1.2. Blood Samples from ARDS Cases and Controls

From the Molecular Epidemiology of ARDS (MEARDS) prospective cohort study [34,35], 160 Acute Respiratory Distress Syndrome (ARDS) cases and 142 controls were recruited for RNA-Seq analysis of blood samples. RNA was extracted by PAXgene Blood RNA Kit (QIAGEN LLC—USA, Germantown, MD, USA) and selected by using oligo(dT) beads. Sequencing libraries were built using MGIEasy RNA Library Prep Kit (MGI Tech Co., Ltd., Shenzhen, China) and subsequently sequenced on the MGISEQ-2000 platform. The 100 bp pair-end sequencing reads were filtered to remove low-quality, adaptor-polluted and high content of unknown base (N) reads. Further, sequencing reads were mapped to reference genome GRCh38 using STAR [36] and counts of reads aligning to known genes were generated by featureCounts [37]. Quality controls were performed for evenness of coverage, rRNA content, genomic context of alignments, as described in Du et al. [35]. Then, limma-voom [38] was used to perform whole-transcriptome differential expression analysis from raw counts.

#### 4.1.3. TCGA Lung Adenocarcinoma Tumors

The data retrieval and processing followed our previous study [39]. Reads per kilobase per million mapped reads (RPKM) values were calculated for association analysis.

### 4.2. Single-Cell Transcriptomics

We analyzed scRNA-seq datasets of nasopharynx/pharynx, BALF and PBMC samples from COVID-19 patients and healthy controls. The nasopharynx/pharynx data were available in the study of Chua et al. [40] with the count matrix and cell type identification derived from 11 moderate and eight critical COVID-19 patients and five healthy controls. The count data of BALF samples from six severe and three mild COVID-19 patients as well as three healthy controls were downloaded from GEO GSE145926 [14]. Also, the count data from PBMC samples of six severe, four mild, and one asymptomatic COVID-19 patients and four healthy controls were downloaded from GEO GSE149689 of Lee et al. [16]. In addition, we analyzed a second PBMC scRNA-seq dataset from the study of Wilk et al. [15], which was from six healthy controls and seven COVID-19 patients. Four of eight COVID-19 samples were collected from patients who were diagnosed with ARDS and were ventilated. Two samples were collected from one patient in the non-severe condition without ventilation and in the severe condition with ventilation.

For each dataset, sequencing read counts in single cells were processed and analyzed using the Seurat 3.0 package [41]. According to “nFeature_RNA” (the total number of genes detected in each cell) and “percent.mt” (the proportion of transcripts that are of mitochondrial origin in each cell), data were filtered to remove doublets, dead cells and empty droplets. We applied “nFeature_RNA > 350 & nFeature_RNA < 11000 & percent.mt < 10” for the nasopharynx/pharynx dataset, “nFeature_RNA > 350 & nFeature_RNA < 6500 & percent.mt < 10” for the BALF dataset, “nFeature_RNA > 350 & nFeature_RNA < 6000 & percent.mt < 20” for the Lee PBMC dataset, and “nFeature_RNA > 350 & nFeature_RNA < 3500 & percent.mt < 15” for the the Wilk PBMC dataset. After filtering, 110,145 cells including 9326 DCs were left for further analysis in the nasopharynx/pharynx dataset, 71,415 cells including 1484 DCs were left in the BALF dataset, 58,187 cells including 526 DCs were left in the Lee et al. [16] PBMC dataset, and 36,925 cells including 514 DCs were left in the Wilk PBMC dataset. Sequentially, data were normalized using the “LogNormalize” method, 2000 highly variable feature were selected, data were centered and scaled by their root mean square, and PCA analysis were performed for dimension reduction. Further, the Louvain optimization-based clustering method was used on the top 20 principal components to identify clusters, which were visualized in reduced dimensions of t-distributed stochastic neighbor embedding (tSNE) and Uniform Manifold Approximation and Projection (UMAP). Cell types in the nasopharynx/pharynx dataset and the Wilk et al. PBMC dataset were identified by their original studies. Following the approaches used in the study of Wilk et al. [15], we used SingleR [42] to identify cell types for cell clusters detected in the BALF and Lee et al. [16] PBMC datasets and validated them by markers used in their original studies respectively (Supplementary Figure S13). In the nasopharynx/pharynx samples, 22 cell types were identified including squamous cells, non-resident macrophages (NR macrophages), monocyte-derived macrophages (MoD macrophages), resident macrophages (R macrophages), neutrophils, CD4^+^ T cells, CD8^+^ T cells, B cells, ciliated cells, NK cells, NKT cells, differentiating ciliated cells (Diff ciliated), DCs, IFNG-responsive cells (IRC), basal cells, secretory cells, differentiating secretory cells (Diff secretory), outliers epithelial cells (Out epithelial), ionocytes, Unknown epithelial cells, mast cells and FOXN4^+^ cells. In the BALF samples, we identified CD8^+^ T cells, CD4^+^ memory T cells (CD4m T), gamma delta T cells (gd T), alveolar macrophages (A macrophages), MoD macrophages, epithelial cells, DCs, NK cells, plasma B cells (PB), neutrophils and monocytes. In the Lee PBMC dataset, we identified immature B cells, naive B cells, CD4m T cells, CD8^+^ T cells, CD4^+^ T cells, erythroblasts, gd T cells, CD16^+^ monocytes, CD16^−^ monocytes, platelets, NK cells, DCs and intermediate monocytes (Int monocytes). In the Wilk PBMC dataset, 20 cell types were identified, including activated granulocytes (Act granulocytes), B cells, CD14^+^ monocytes, CD16^+^ monocytes, CD4^+^ T cells, CD4m T cells, naive CD4^+^ T cells (CD4n T), effector CD8^+^ T cells (CD8eff T), CD8m T cells, class-switched B cells (Switched B), DCs, gd T cells, IgA-producing PB cells (IgA PB), IgG-producing PB cells (IgG PB), neutrophils, NK cells, DCs, platelets, red blood cells (RBC), as well as stem cells and eosinophils (SC & Eosinophil). To compare gene expression between cell types, Wilcoxon rank sum test was used.

Within identified DC cells in each dataset, we re-clustered cells to identify DC subsets following the same protocol as above. We used well evidenced markers for cDC1 (*CLEC9A*, *C1orf54*, *HLA-DPA1*, *CADM1*, *CAMK2D*, *XCR1*), cDC2 (*CD1C*, *FCER1A*, *CLEC10A*), pDC (*DAB2*, *GZMB*, *JCHAIN*, *SERPINF1*, *ITM2C*, *CLEC4C*, *LILRA4*), mature DC (*LAMP3*, *CD86*, *CD83*, *CD40*, *ICAM1*, *ITGA4*, *CCL8*, *CCL22*, *CCL17*, *CXCL8*, *CXCL9*, *CXCL10*, *CXCL11*, *CCR7*), macrophage-like DC (*S100A9*, *S100A9*, *S100A8*, *VCAN*, *LYZ*, *CD14*), AXL^+^ DC (*ANXA1*, *AXL*, *PPP1R14A*, *SIGLEC6*, *CD22*) and non-classical DC (*FCGR3A*, *CX3CR1*). To evaluate the similarity of DC subtypes identified in above scRNA-seq datasets, we integrated these datasets using Seurat and visualized the integrated matrix with UMAP. Monocle [43] and TSCAN [44] were used to infer the single cell trajectory.

### 4.3. Group Comparison and Association Testing

Linear regression was used to evaluate the association between variables and perform two-group comparison. To evaluate the association of *CD209* expression with tumor stage and survival of lung adenocarcinoma tumors, ordinal regression and Cox proportional hazard model were used respectively. Tumor vs. normal paired differential expression analysis were performed using paired *t*-test.

### 4.4. Pathway and Network Analyses

Pathway and network analyses were performed to systematically investigate biological processes involved in COVID-19. We calculated transcriptome-wide fold changes (FC) of expression and ranked the differential expression according to log_2_FC. Based on these ranks, pre-ranked Gene Set Enrichment Analysis (GSEA) [17] was applied to evaluate the enrichment of Kyoto Encyclopedia of Genes and Genomes (KEGG) [45] pathways. Further, the summary statistics of GSEA analysis were used to build connection networks of detected pathways by the Enrichment Map implemented in Cytoscape [46]. Pathways with *q*-value < 0.2 were included into networks and the connectivity strength was determined by an edge cutoff as 0.25. In addition, networks of genes with absolute value of the average of log_2_FC >1 were constructed using the Cytoscape StringAPP [47].

### 4.5. Inference and Comparison of Transcriptional Bursting Kinetics between DC Subsets

To infer the bursting kinetics from single cell transcriptomic data, we developed a Bayesian hierarchical framework to estimate the bursting kinetic parameters (i.e., transcribe rate s, activation rate $k_{on}$ and deactivation rate $k_{off}$). In the spirit of the conventional Poisson-Beta model [48], our method considered the transcriptional bursting process characterized by burst size $s/k_{off}$ and burst frequency $k_{on}$. A major limitation of Poisson-Beta model is that it neglects the remarkable noisy nature of sequencing technology [49,50,51], which usually leads to overestimation of the dispersion and consequently jeopardize the validity to infer bursting kinetics. To address this issue, we reconstructed the mean-variance relationship by utilizing the Generalized additive model (GAM) that can resist and down-weight the effect of a small number of atypically high dispersion estimates especially for genes with few counts. The re-estimated dispersion parameter ϕ based on the re-fitted mean-variance function was then incorporated into the framework, assuming that the sampling distribution of the underlying mean λ is gamma. Specifically, let $y∼Poisson\left(\lambda \right)$ denotes the observed number of mRNA molecules:

(1) $$\lambda ∼gamma\left(1/\phi^{2},sp\phi^{2}\right)$$

(2) $$p|k_{on},k_{off}∼Beta\left(k_{on},k_{off}\right)$$

where *p* denotes the fraction of time that a gene spends in the active state. With the proper choices of the priors and hyperpriors, the posterior parameters were estimated via the Markov Chain Monte Carlo (MCMC) algorithm implemented in the R package BISC (https://github.com/thecailab/BISC, accessed on 5 October 2020).

We also formulated a differential bursting analysis framework that can further explored the change in bursting kinetics between different study conditions. After applying the MCMC method, the MCMC samples provided various representative combinations of bursting frequency and size values, while the credible differences of bursting frequency and size could be examined by computing and summarizing the changes at each combination of parameters values. Those credible differences could then be used to assess the credibility of “null value” [52].

We applied the proposed method to investigate the differences of bursting kinetics in ANXA1^+^ DCs in COVID-19 lung compared to pDCs and cDC2s separately. With the processed scRNA-seq count data, changes in bursting frequency and size estimates (in log_2_ scale) were computed.

### 4.6. Transcriptome-Based Phenome-Wide Association Study

We utilized public resources to search for CD209 related traits/disease candidate. PhenoScanncer [53] was used to obtain associated phenotypes from genome-wide association study (GWAS) studies. Also, PhenomeXcan [54] underlying phenome-wide association study (PheWAS) analytic framework was used to infer associations of CD209 lung expression and multiple traits, by integrating GWAS summary statistics of 4091 traits and Genotype-Tissue Expression data from GTEx [55] lung tissue version 8.

### 4.7. Mendelian Randomization Analysis

Mendelian Randomization (MR) analysis provides evidence for a causal relationship between exposure and outcome. To infer the causal effect of plasma expression of ACE2 and DC-SIGN on COVID-19 risk and severity, we applied the TwoSampleMR R package [56] for MR analysis. For exposures of protein expression of SARS-CoV-2 receptors, we obtained publicly available plasma protein QTL summary statistics from the study of Sun et al. [57]. For outcomes of COVID-19 risk and severity, we separately required GWAS summary statistics from GRASP (https://grasp.nhlbi.nih.gov/downloads/COVID19GWAS/06052020/UKBB_covid19_ALLwhites_060520.SAIGE.bgen.txt.gz, accessed on 31 July 2020) for COVID-19 risk, and that from the study of Ellinghaus et al. [58] for COVID-19 severity. Independent genetic instruments of exposures were selected for MR analysis based on strict criteria of minor allele frequency (MAF) > 0.05, linkage disequilibrium (LD) *r*^2^ < 0.01 within a clumping window of 10,000 kb, and beyond genome-wide significance (*p* < 5 × 10^−8^). As there were no genetic instruments of plasma ACE2, the significance threshold for selecting instruments was set as *p* < 10^−4^. Ultimately, we found 3, 60, and 1 single nucleotide polymorphisms (SNPs) as independent genetic instruments for plasma CD209, ACE2, and CLEC4M, respectively. Inverse-variance-weighted (IVW), weighted median, and MR-Egger regression methods were used to calculate effect size (β) and corresponding standard error (SE). The Wald ratio method was used if only one genetic instrument remained. Heterogeneity was estimated using MR-Egger and IVW methods. Directional pleiotropy was estimated via MR-Egger intercept test.

### 4.8. Software

All data management, statistical analyses and visualizations were accomplished using R software version 4.0.2 (R Core Team, 2020).

## Data Availability

Four processed datasets from the DCs of nasopharynx/pharynx, BALF and PBMC samples from COVID-19 patients and healthy people were available for visualization and exploration in SCANNER [59] (https://www.thecailab.com/scanner/, accessed on 21 May 2021).

## Supplementary Materials

The following are available online at https://www.mdpi.com/article/10.3390/ijms22179228/s1.
