# Supplementary material for: SARS-CoV-2 Impairs Dendritic Cells and Regulates DC-SIGN Gene Expression in Tissues
Source: Int J Mol Sci. 2021 Aug 26;22(17):9228. doi: 10.3390/ijms22179228 (PMC8431536; doi:10.3390/ijms22179228)
Supplement: Supplementary file 1 [file ijms-22-09228-s001.zip › ijms-1310048-supplementary.pdf]

Supplementary Materials

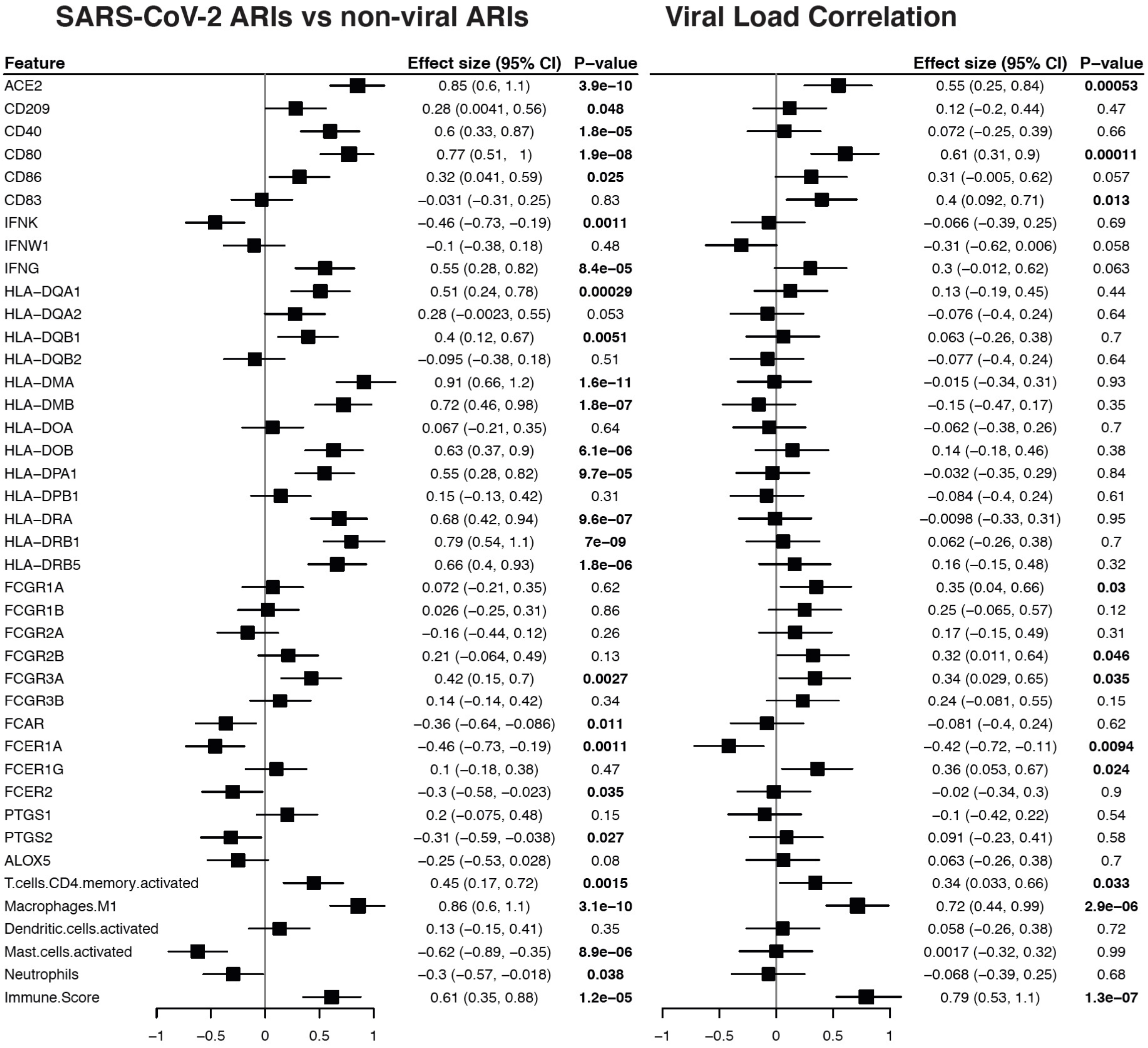

Figure S1. The association of SARS-CoV-2 receptors and immune factors with SARS-CoV-2 infection and viral load in nasopharynx/pharynx samples of ARIs. For each feature, results of comparisons between SARS-CoV-2 ARIs and non-viral ARIs are shown on the left and correlations with SARS-CoV-2 viral load are shown on the right. The estimated coefficient and 95% confidence intervals (CIs) were plotted. Significant P values which are less than 0.05 are shown in bold.

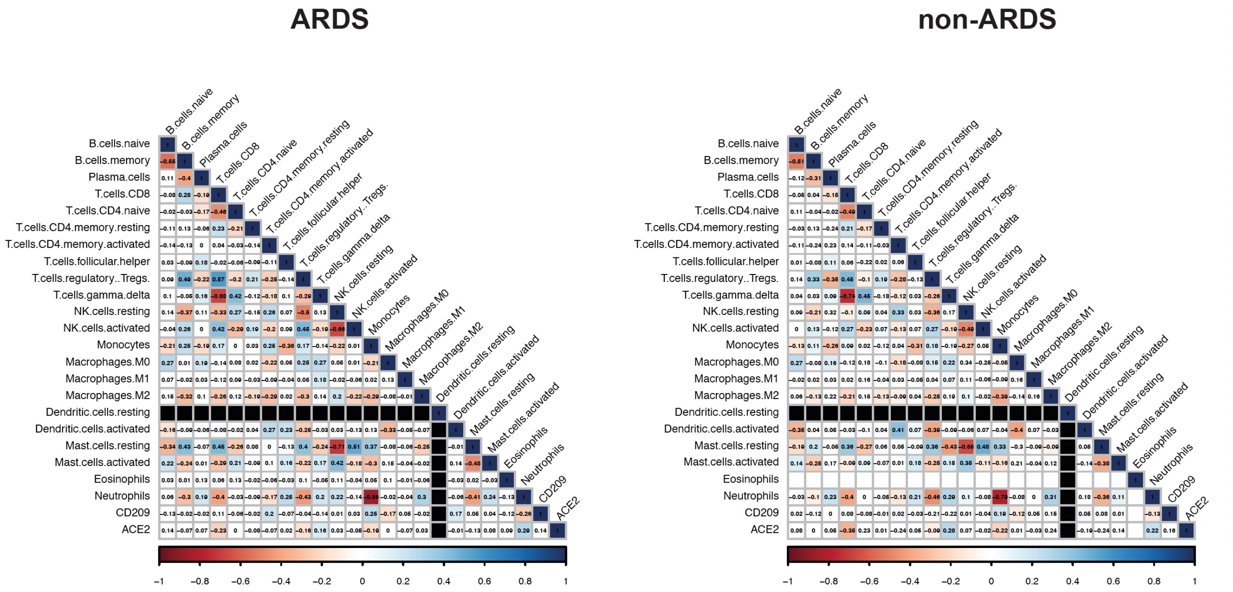

Figure S2. Correlation matrix among immune cell composition and the expression of *ACE2*, *CLEC4M* and *CD209* in ARDS and non-ARDS blood samples. Pairwise similarity (Spearman correlation) among the expression of candidate genes and 22 immune cells proportions in ARDS blood samples are shown on the left, and that in non-ARDS blood samples are shown on the right.

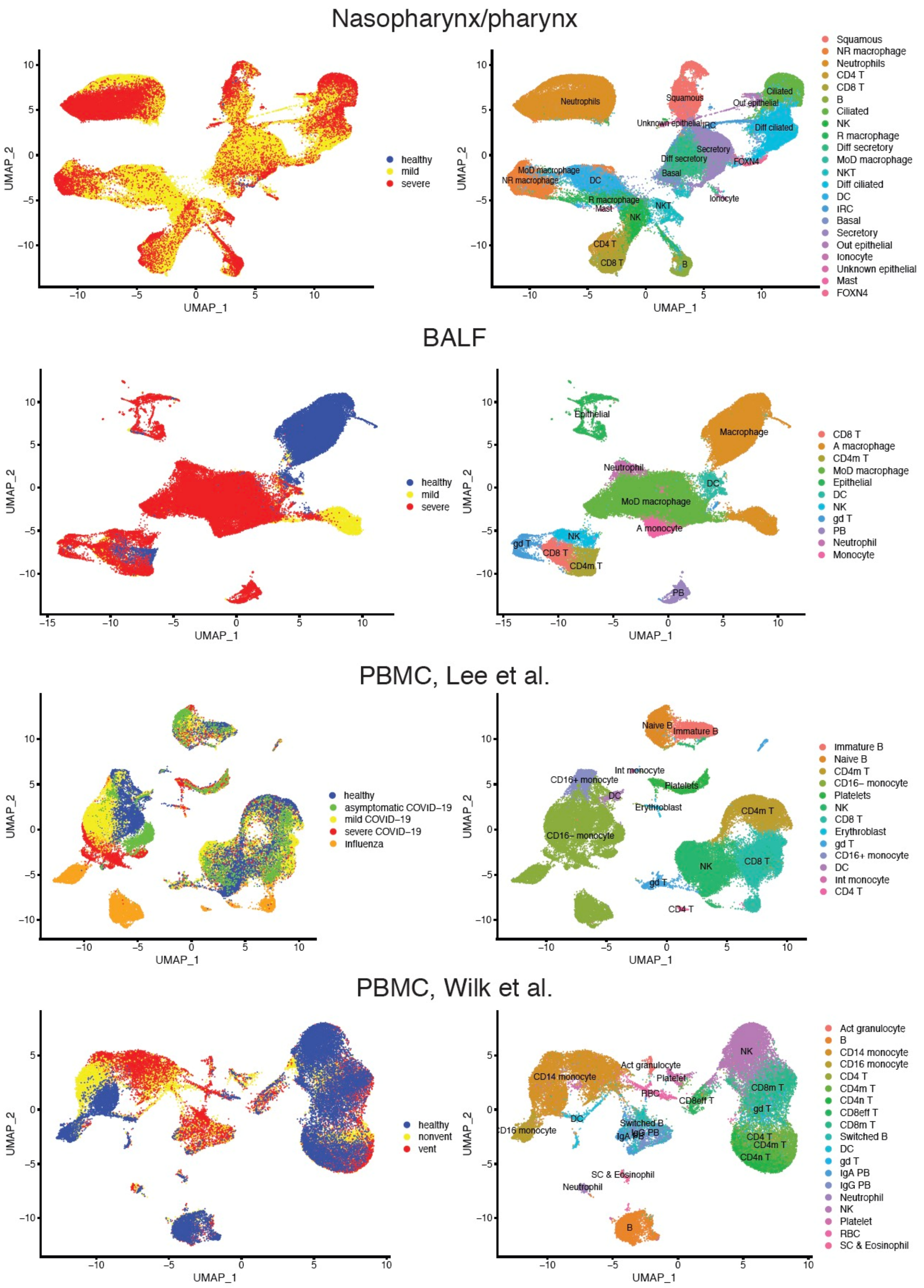

Figure S3. Clusters of single-cell transcriptomics of nasopharynx/pharynx, BALF and PMBC samples from COVID-19 cases and healthy controls. For each dataset, the left shows UMAP embedding of single-cell transcriptome profiles from COVID-19 mild and severe cases, and healthy controls. The right shows identified cell types.

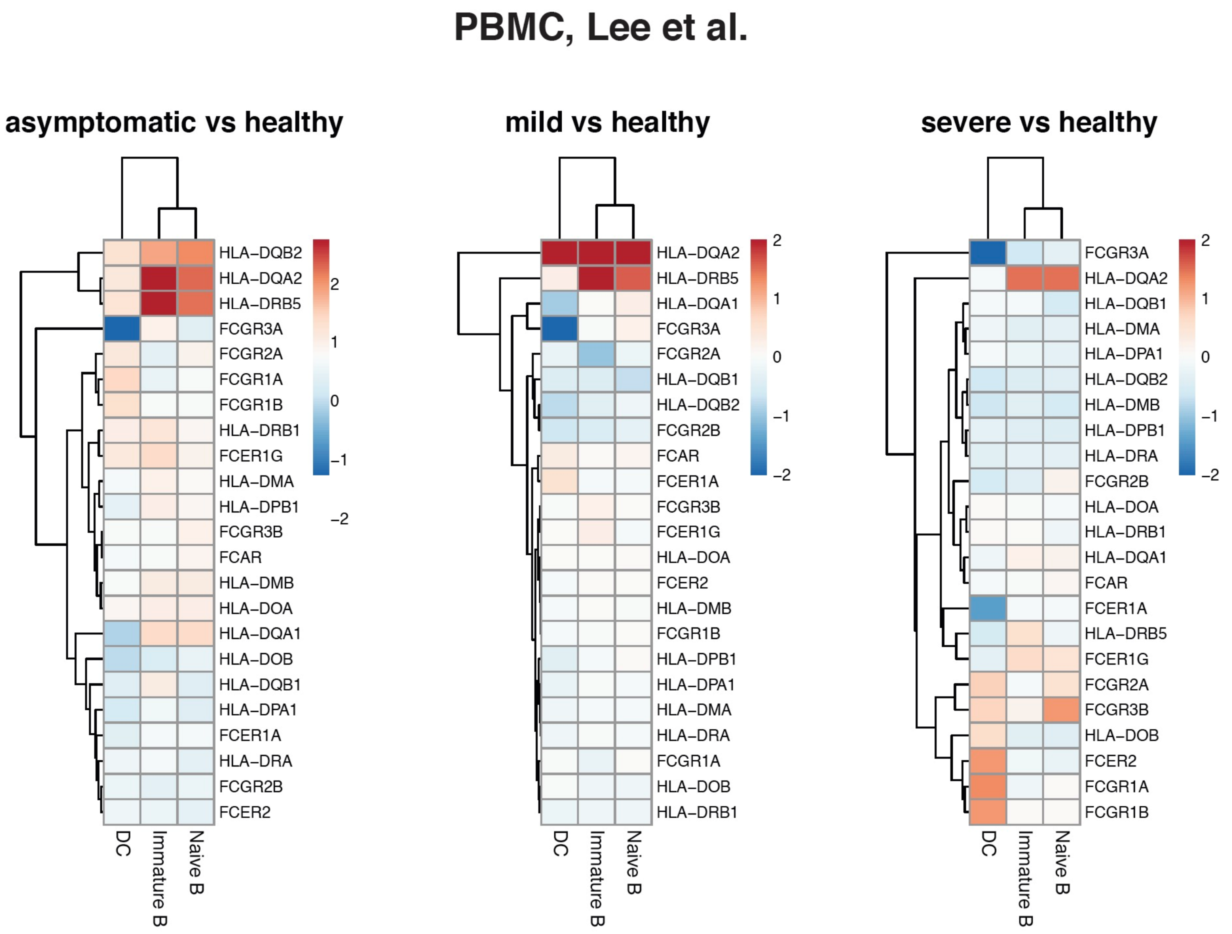

Figure S4. The dysregulation of gene expression of MHCII and FcRs in APCs in blood with COVID-19. In the PBMC scRNA-seq dataset of Lee et al., the alterations of gene expression in asymptomatic, mild and severe COVID-19 cases compared to healthy controls were analyzed and visualized in heatmaps. Red indicates upregulation and blue indicates downregulation. The scale corresponds to log_2FC.

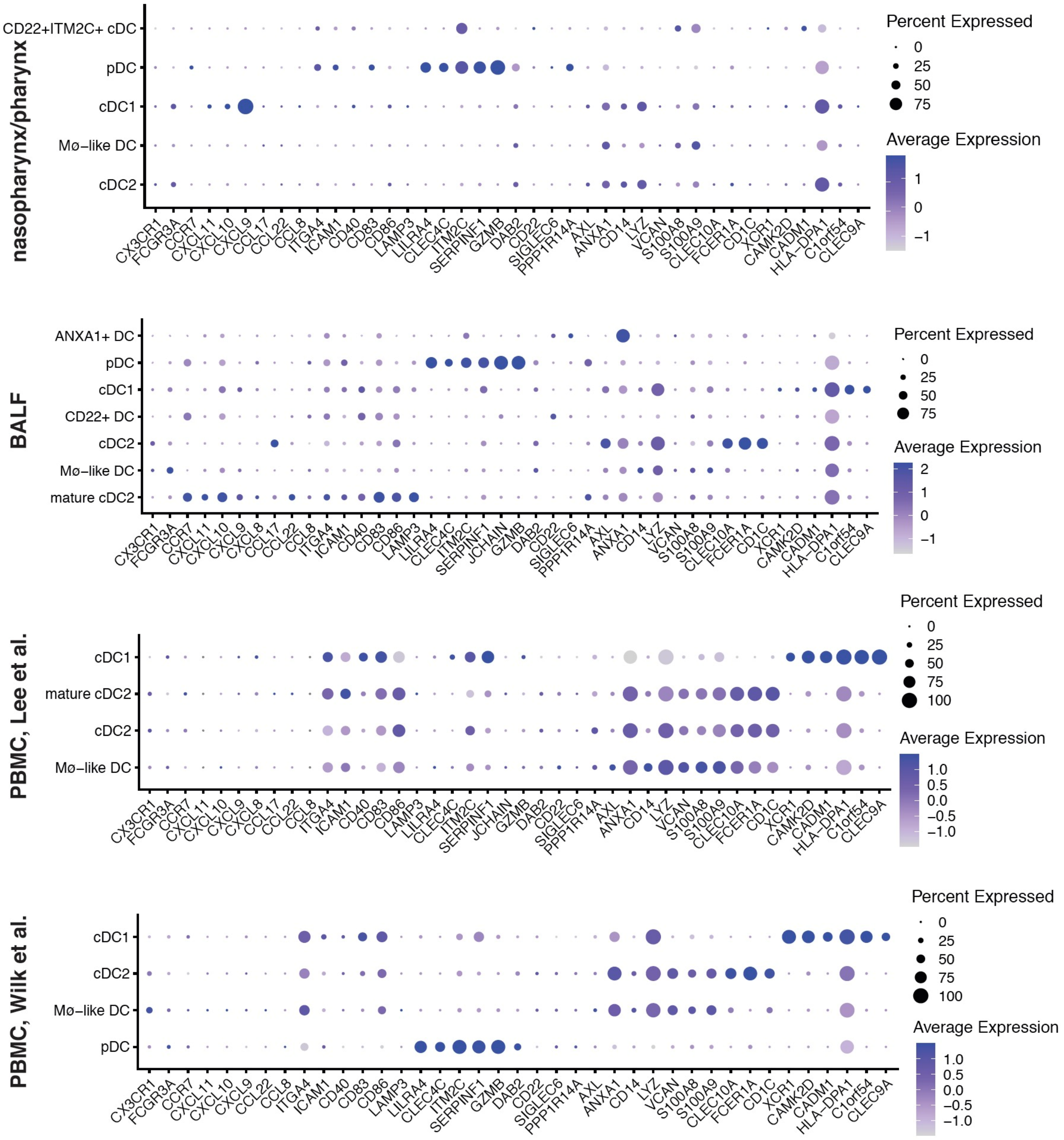

Figure S5. Expression profiles of DC subset markers in four scRNA-seq datasets of nasopharynx/pharynx, BALF and PBMC samples from COVID-19 patients and healthy controls. For each dataset, the detection rate (corresponding to dot size, larger dot indicates higher detection rate) and average expression level (corresponding to dot color, darker indicates higher expression level) of each cell-type marker are shown in identified DC subtypes.

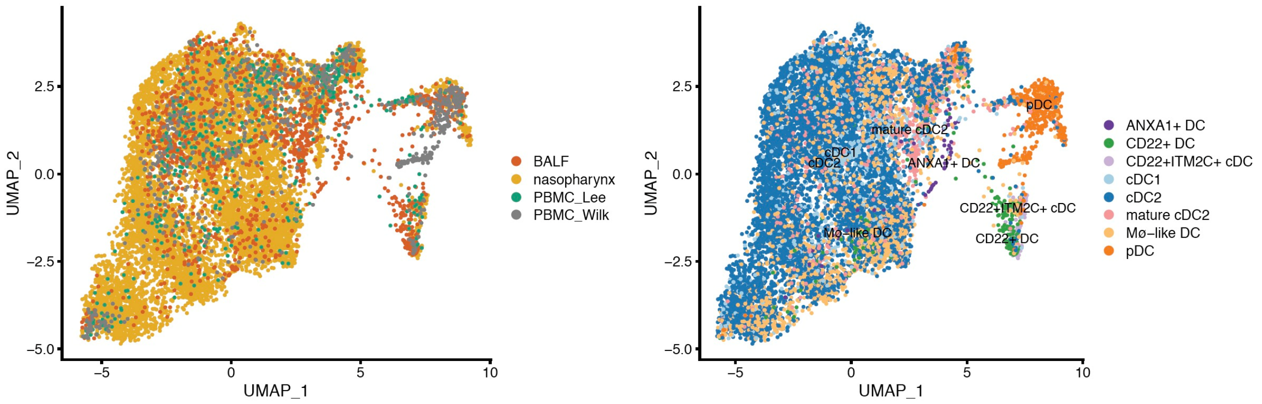

Figure S6. Integrated single-cell transcriptomics of nasopharynx/pharynx, BALF and PMBC samples from COVID-19 cases and healthy controls. The left shows UMAP embedding of single-cell transcriptome profiles from COVID-19 mild and severe cases, and healthy controls. The right shows identified cell types.

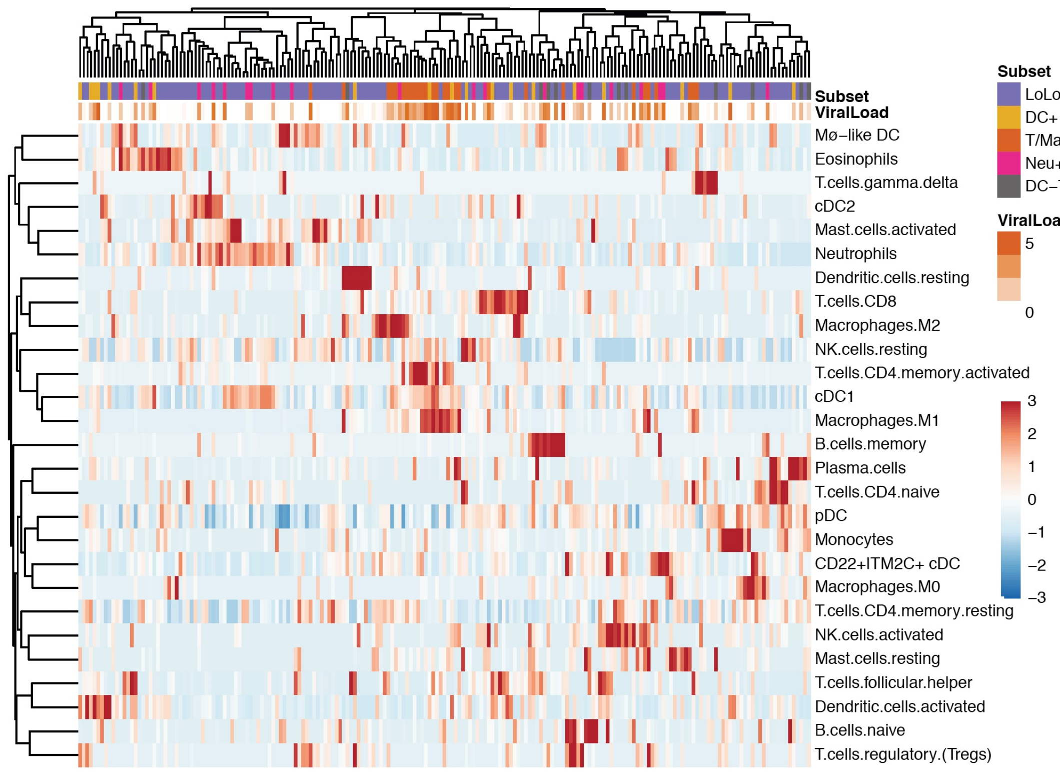

Figure S7. Hierarchical clustering and heatmap of immune cell composition in ARI nasopharynx/pharynx samples from COVID-19 cases and healthy controls. For each cell type, red indicates relatively higher proportion and blue indicates relatively lower proportion across samples. The scale corresponds to the relative value across samples.

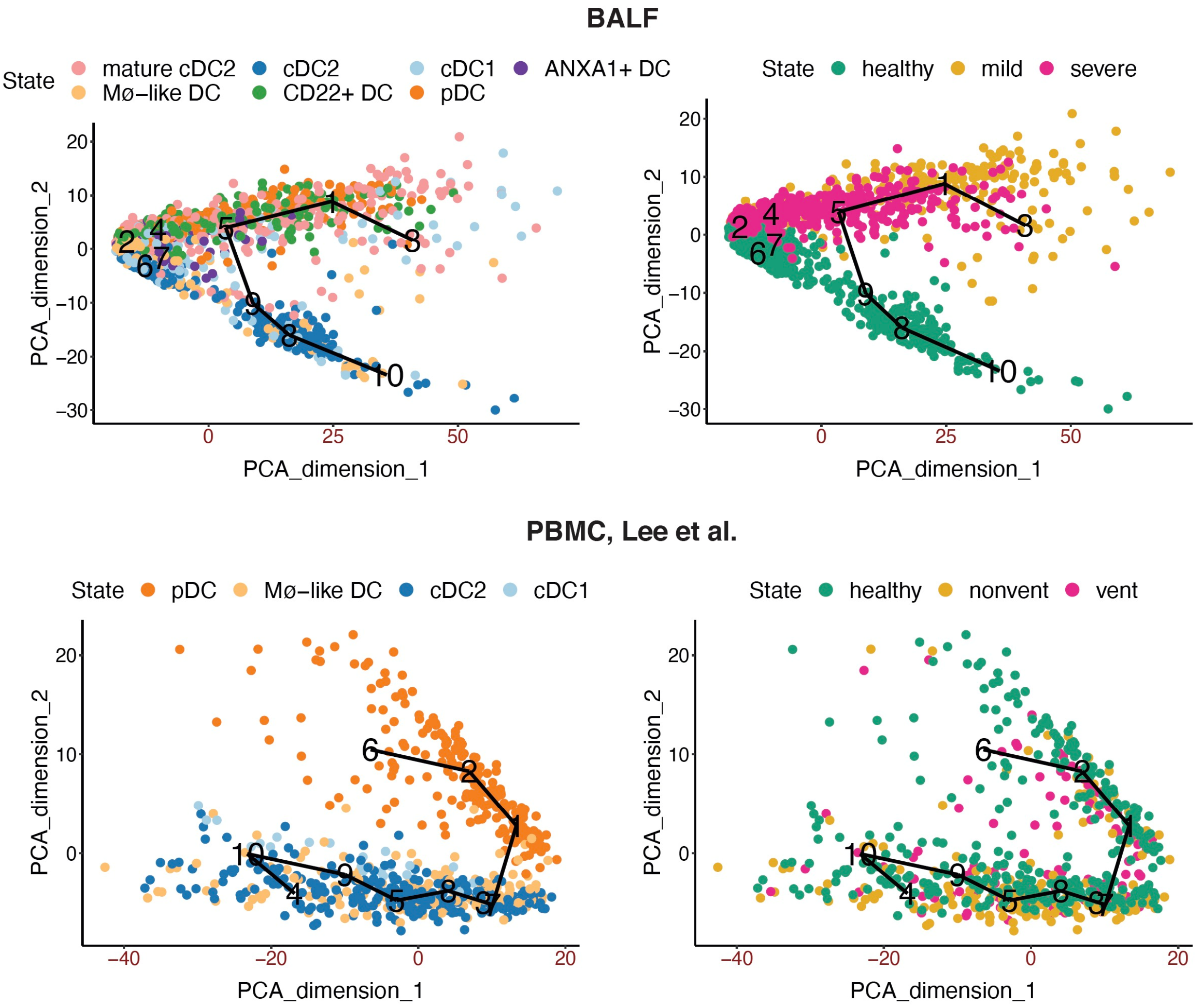

Figure S8. Single-cell trajectory of DCs in COVID-19 BALF and PMBC samples. The Single-cell trajectory was inferred by TSCAN. The left shows ordered cells labeled with cell types. The right shows ordered cells from COVID-19 mild and severe cases, and healthy controls.

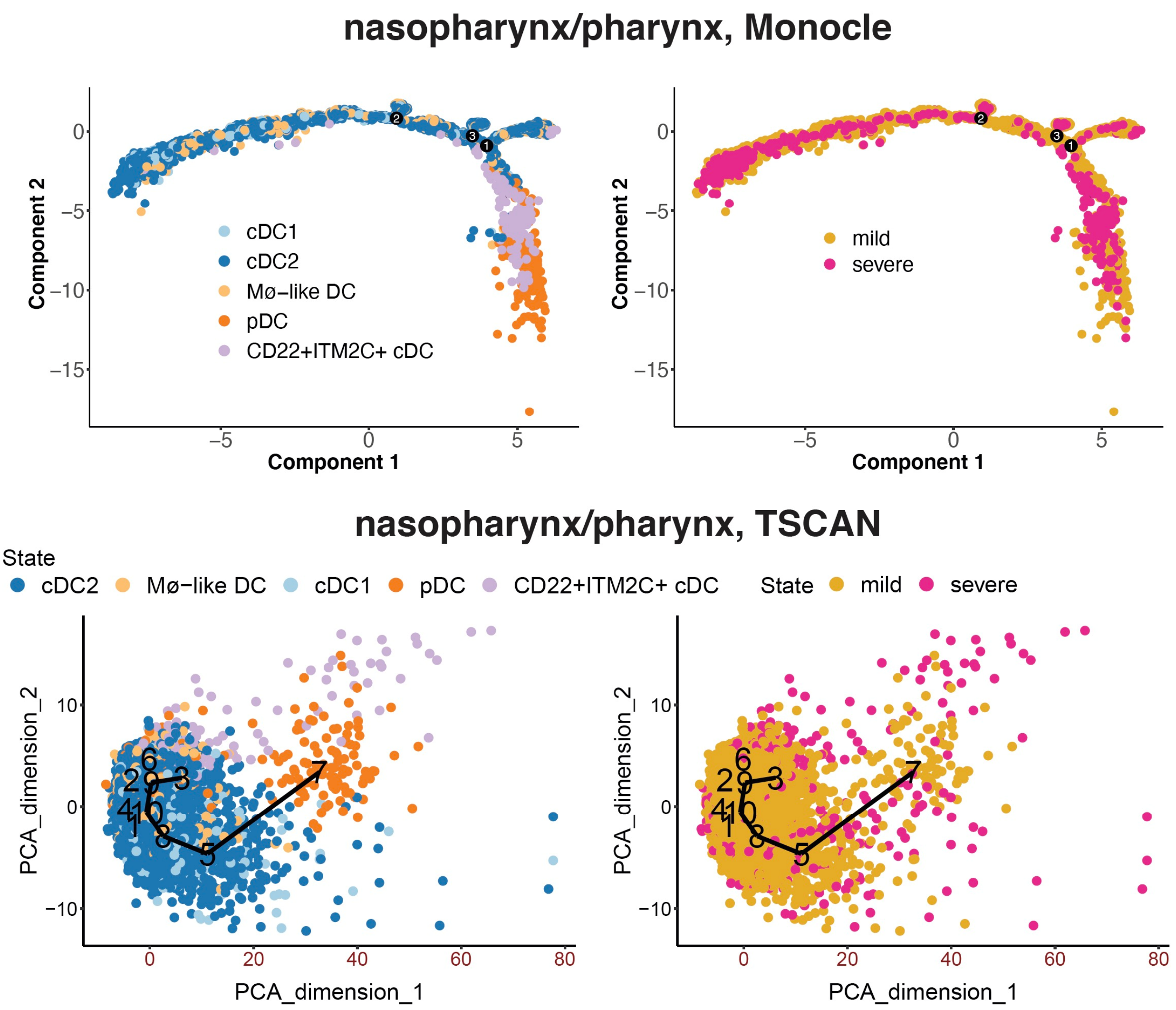

Figure S9. Single-cell trajectory of DCs in COVID-19 nasopharynx/pharynx samples. The Single-cell trajectory was inferred by Monocle (top) and TSCAN (bottom). The left shows ordered cells labeled with cell types. The right shows ordered cells from COVID-19 mild and severe cases.

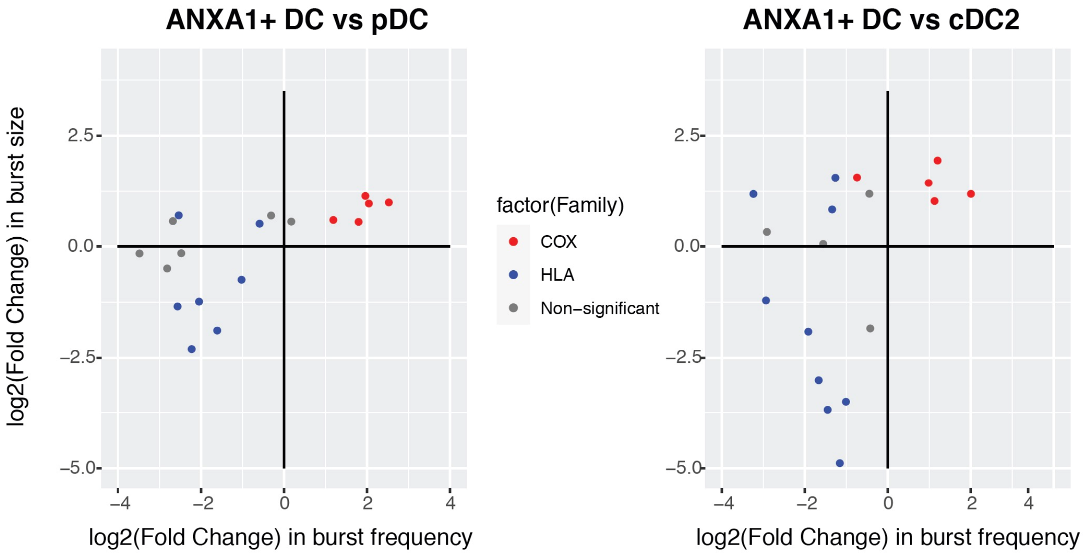

Figure S10. Transcriptional bursting profiles in different DC subsets in lung. The changes in burst frequency and burst size of HLA genes (blue) and Cox genes (red) in ANXA1+ DC compared to pDC (left) and cDC2 (right) are plotted in $\log_2$ scale.

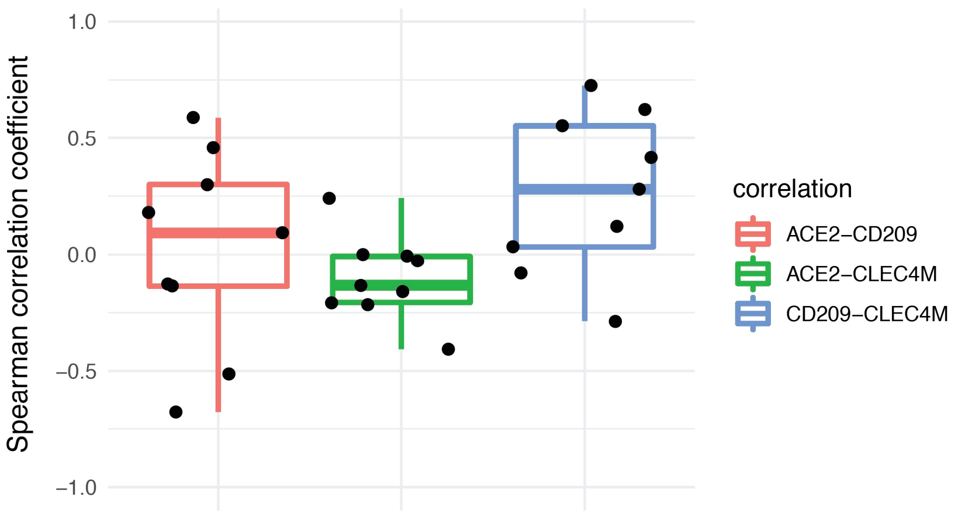

Figure S11. Correlation of genes *ACE2*, *CLEC4M* and *CD209* in normal lung tissues. Spearman correlation coefficients for expression of each pair of genes were shown in boxplot.

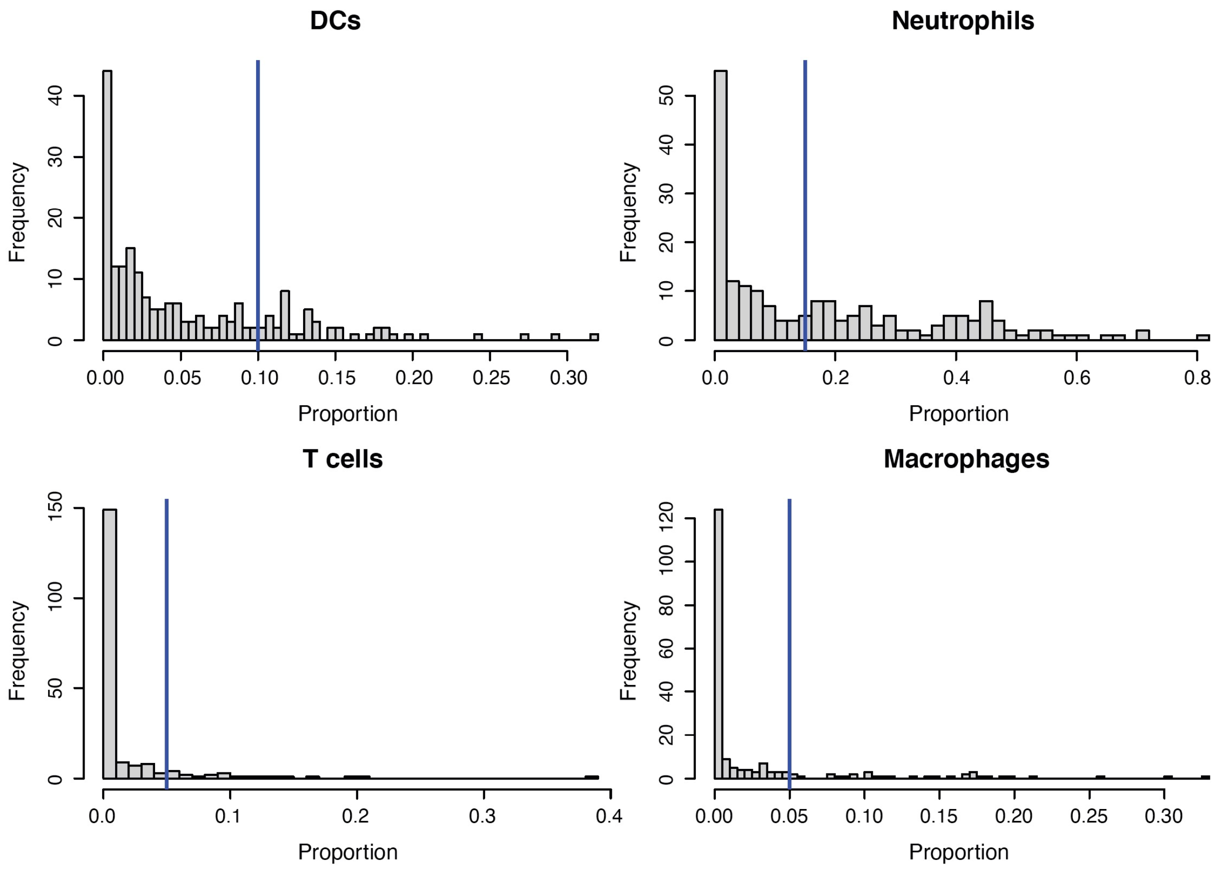

Figure S12. Distribution of cell proportions in ARI samples. The distribution of inferred cell proportions of DCs, Neutrophils, T cell and Macrphages were shown in histograms. Blue verticle lines show the thresholds for identify subtypes with enrichement of specific cell types.

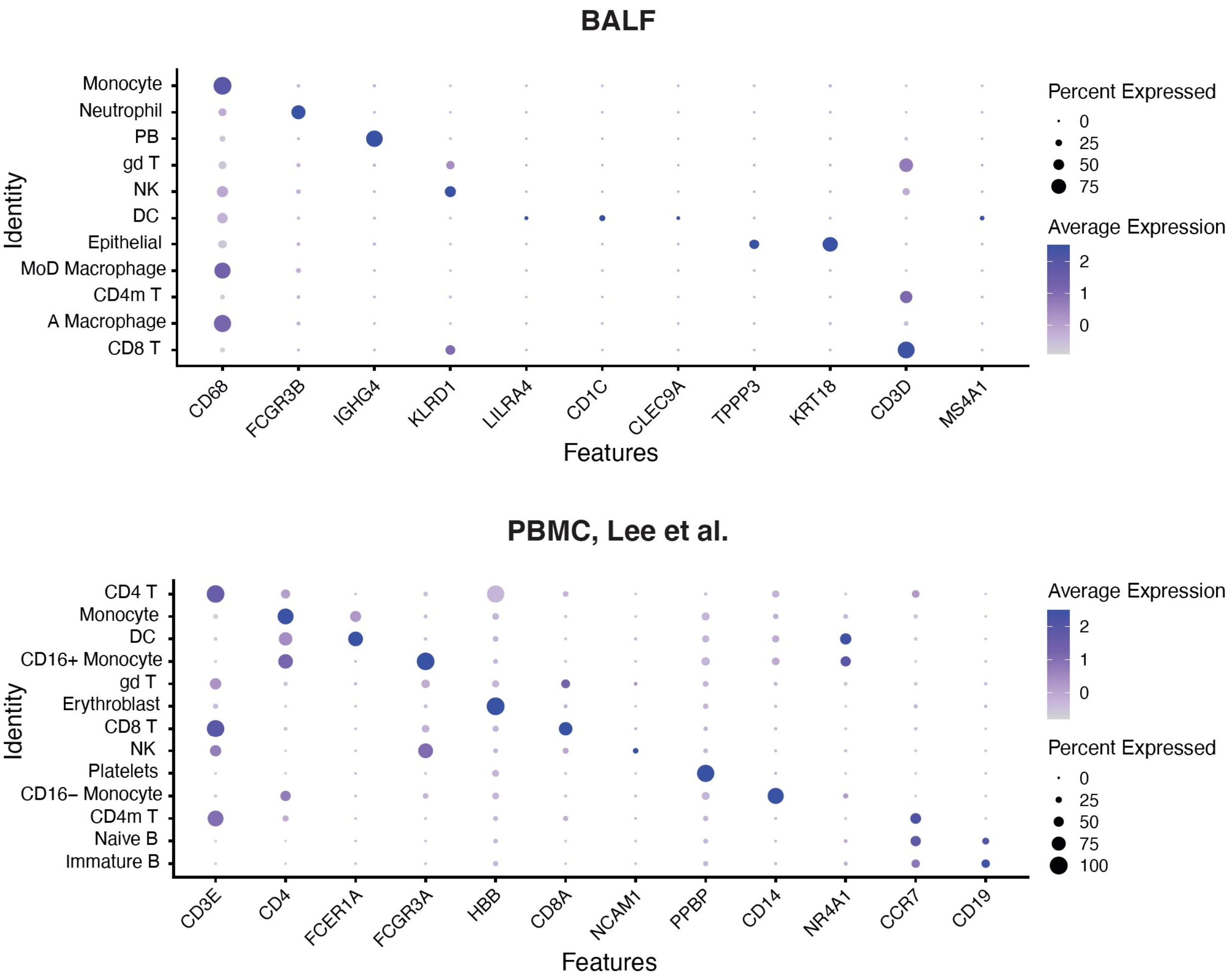

Figure S13. Expression profiles of cell type markers in BALF and Lee et al. PBMC samples. For each dataset, the detection rate (corresponding to dot size, larger dot indicates higher detection rate) and average expression level (corresponding to dot color, darker indicates higher expression level) of each cell-type marker are shown in identified cell types.

Table S1. Mendelian Randomization inferred causal effect of plasma expression of ACE2, DC-SIGN and L-SIGN on COVID-19 risk and severity in the European population.

Outcome	Exposure	Method	nsnp	b	se	pval
COVID-19 risk	plasma DC-SIGN expression	MR Egger	3	0.12	0.10	4.49×10^{-1}
		Weighted median	3	0.14	0.05	4.17×10^{-3}
		Inverse variance weighted	3	0.14	0.05	3.39×10^{-3}
		Simple mode	3	0.11	0.07	2.59×10^{-1}
		Weighted mode	3	0.13	0.05	1.21×10^{-1}
COVID-19 severity		MR Egger	3	0.42	0.18	2.61×10^{-1}
		Weighted median	3	0.31	0.06	2.21×10^{-7}
		Inverse variance weighted	3	0.30	0.08	5.41×10^{-5}
		Simple mode	3	0.35	0.13	1.14×10^{-1}
		Weighted mode	3	0.34	0.07	3.60×10^{-2}
COVID-19 risk	plasma ACE2 expression	MR Egger	60	0.38	0.21	8.41×10^{-2}
		Weighted median	60	0.04	0.07	6.08×10^{-1}
		Inverse variance weighted	60	0.04	0.05	4.05×10^{-1}
		Simple mode	60	0.04	0.15	8.09×10^{-1}
		Weighted mode	60	0.04	0.16	8.24×10^{-1}
COVID-19 severity		MR Egger	59	0.28	0.26	2.86×10^{-1}
		Weighted median	59	0.05	0.09	6.08×10^{-1}
		Inverse variance weighted	59	0.01	0.06	9.02×10^{-1}
		Simple mode	59	0.15	0.22	5.01×10^{-1}
		Weighted mode	59	0.17	0.24	4.70×10^{-1}
COVID-19 risk	plasma L-SIGN expression	Wald ratio	1	0.41	0.23	7.70×10^{-2}
COVID-19 severity		Wald ratio	1	−0.18	0.29	5.42×10^{-1}

nsnp: number of SNPs used in analysis; b: beta coefficient; se: standard error

Table S2. The top 100 CD209 expression associated traits identified by GWAS.

rsid	a1	a2	Phenotype	phenotype_source	ancestry	effect_size	pvalue
rs536227604	T	C	Cause of death: asthma, unspecified	UKBB	European	−0.067	1.00×10^{-13}
rs538085590	A	G	Benign neoplasm of urinary organs	UKBB	European	−0.002	2.85×10^{-12}
rs71534255	A	C	Cause of death: chronic myeloid leukaemia	UKBB	European	0.051	1.01×10^{-11}
rs536227604	T	C	Malignant neoplasm of tonsil	UKBB	European	−0.004	8.42×10^{-10}
rs73493971	A	G	Self-reported parathyroid hyperplasia or adenoma	UKBB	European	−0.003	4.40×10^{-9}
rs190601505	A	G	Self-reported parathyroid hyperplasia or adenoma	UKBB	European	0.003	9.58×10^{-9}
rs73493971	A	G	Cause of death: cerebrum, except lobes and ventricles	UKBB	European	−0.027	1.07×10^{-8}
rs190601505	A	G	Cause of death: cerebrum, except lobes and ventricles	UKBB	European	0.026	1.48×10^{-8}
rs185373670	T	C	Treatment with cinnarizine	UKBB	European	0.004	2.32×10^{-8}
rs11465395	C	G	Treatment with maxalt 5mg tablet	UKBB	European	0.003	2.99×10^{-8}
rs73493973	A	G	Treatment with maxalt 5mg tablet	UKBB	European	0.003	3.94×10^{-8}
rs73493968	T	C	Treatment with maxalt 5mg tablet	UKBB	European	−0.003	4.09×10^{-8}
rs73493965	T	C	Treatment with maxalt 5mg tablet	UKBB	European	0.003	4.22×10^{-8}
rs73493966	C	G	Treatment with maxalt 5mg tablet	UKBB	European	−0.003	4.30×10^{-8}
rs114437818	T	G	Treatment with maxalt 5mg tablet	UKBB	European	−0.003	4.40×10^{-8}
rs114878389	A	G	Treatment with maxalt 5mg tablet	UKBB	European	0.003	4.41×10^{-8}
rs112039104	A	C	Treatment with maxalt 5mg tablet	UKBB	European	0.003	4.69×10^{-8}
rs113398041	T	C	Treatment with maxalt 5mg tablet	UKBB	European	−0.003	4.71×10^{-8}
rs73493975	A	G	Treatment with maxalt 5mg tablet	UKBB	European	−0.003	4.81×10^{-8}
rs61058460	T	C	Treatment with maxalt 5mg tablet	UKBB	European	−0.003	5.45×10^{-8}
rs77982873	T	C	Treatment with maxalt 5mg tablet	UKBB	European	−0.003	5.49×10^{-8}
rs77377087	A	T	Treatment with maxalt 5mg tablet	UKBB	European	−0.003	5.49×10^{-8}
rs61468896	T	C	Treatment with maxalt 5mg tablet	UKBB	European	0.003	5.52×10^{-8}
rs17159887	T	C	Treatment with maxalt 5mg tablet	UKBB	European	−0.003	6.28×10^{-8}
rs57889796	T	C	Treatment with maxalt 5mg tablet	UKBB	European	−0.003	6.56×10^{-8}
rs57668221	C	G	Treatment with maxalt 5mg tablet	UKBB	European	−0.003	6.91×10^{-8}
rs78866372	A	G	Cause of death: cerebral infarction, unspecified	UKBB	European	0.036	7.18×10^{-8}
rs185373670	T	C	Malignant neoplasm of other and unspecified parts of tongue	UKBB	European	0.003	9.50×10^{-8}
rs111716431	A	G	Treatment with maxalt 5mg tablet	UKBB	European	0.003	9.96×10^{-8}
rs536227604	T	C	Self-reported giant cell or temporal arteritis	UKBB	European	−0.003	2.96×10^{-7}
rs185373670	T	C	Enthesopathies of lower limb, excluding foot	UKBB	European	0.003	3.40×10^{-7}
rs538085590	A	G	Cause of death: vascular disorder of intestine, unspecified	UKBB	European	−0.04	4.74×10^{-7}
rs11465411	A	G	Cause of death: inquest adjourned death	UKBB	European	−0.009	8.45×10^{-7}
rs11465410	T	C	Cause of death: inquest adjourned death	UKBB	European	−0.009	8.85×10^{-7}
rs111716431	A	G	Cause of death: urinary tract infection, site not specified	UKBB	European	0.034	9.40×10^{-7}
rs185373670	T	C	Treatment with fybogel orange s or f granules	UKBB	European	0.006	9.52×10^{-7}
rs71534255	A	C	Cause of death: sigmoid colon	UKBB	European	0.087	1.09×10^{-6}
rs11465409	A	G	Cause of death: inquest adjourned death	UKBB	European	0.008	1.11×10^{-6}
rs71534255	A	C	Treatment with metrogel 0.75% gel	UKBB	European	0.003	1.11×10^{-6}
rs11465412	A	G	Cause of death: inquest adjourned death	UKBB	European	0.008	1.25×10^{-6}
rs73493971	A	G	Treatment with cyclizine	UKBB	European	−0.003	1.26×10^{-6}
rs185373670	T	C	Cause of death: secondary malignant neoplasm of retroperitoneum and peritoneum	UKBB	European	0.028	1.65×10^{-6}
rs114878389	A	G	Cause of death: cerebrum, except lobes and ventricles	UKBB	European	0.018	2.39×10^{-6}
rs114437818	T	G	Cause of death: cerebrum, except lobes and ventricles	UKBB	European	−0.018	2.40×10^{-6}
rs11465395	C	G	Cause of death: cerebrum, except lobes and ventricles	UKBB	European	0.018	2.41×10^{-6}
rs73493968	T	C	Cause of death: cerebrum, except lobes and ventricles	UKBB	European	−0.018	2.62×10^{-6}
rs61468896	T	C	Cause of death: cerebrum, except lobes and ventricles	UKBB	European	0.018	2.67×10^{-6}
rs73493966	C	G	Cause of death: cerebrum, except lobes and ventricles	UKBB	European	−0.018	2.69×10^{-6}

rs73493965	T	C	Cause of death: cerebrum, except lobes and ventricles	UKBB	European	0.018	2.69×10^{-6}
rs73493973	A	G	Cause of death: cerebrum, except lobes and ventricles	UKBB	European	0.018	2.73×10^{-6}
rs112039104	A	C	Cause of death: cerebrum, except lobes and ventricles	UKBB	European	0.018	2.80×10^{-6}
rs73493975	A	G	Cause of death: cerebrum, except lobes and ventricles	UKBB	European	−0.018	2.82×10^{-6}
rs113398041	T	C	Cause of death: cerebrum, except lobes and ventricles	UKBB	European	−0.018	2.83×10^{-6}
rs61058460	T	C	Cause of death: cerebrum, except lobes and ventricles	UKBB	European	−0.018	2.85×10^{-6}
rs77982873	T	C	Cause of death: cerebrum, except lobes and ventricles	UKBB	European	−0.018	3.13×10^{-6}
rs77377087	A	T	Cause of death: cerebrum, except lobes and ventricles	UKBB	European	−0.018	3.13×10^{-6}
rs17159887	T	C	Cause of death: cerebrum, except lobes and ventricles	UKBB	European	−0.018	3.15×10^{-6}
rs57668221	C	G	Cause of death: cerebrum, except lobes and ventricles	UKBB	European	−0.018	3.17×10^{-6}
rs57889796	T	C	Cause of death: cerebrum, except lobes and ventricles	UKBB	European	−0.018	3.17×10^{-6}
rs11465395	C	G	Other disorders of ear	UKBB	European	0.002	3.54×10^{-6}
rs71534255	A	C	Cause of death: cardiomegaly	UKBB	European	0.052	3.98×10^{-6}
rs185373670	T	C	Treatment with indoramin	UKBB	European	0.002	4.28×10^{-6}
rs73493968	T	C	Other disorders of ear	UKBB	European	−0.002	4.33×10^{-6}
rs73493973	A	G	Other disorders of ear	UKBB	European	0.002	4.33×10^{-6}
rs73493966	C	G	Other disorders of ear	UKBB	European	−0.002	4.34×10^{-6}
rs111716431	A	G	Self-reported parathyroid hyperplasia or adenoma	UKBB	European	0.002	4.34×10^{-6}
rs538085590	A	G	Diabetes mellitus in pregnancy	UKBB	European	−0.002	4.38×10^{-6}
rs114437818	T	G	Other disorders of ear	UKBB	European	−0.002	4.50×10^{-6}
rs114878389	A	G	Other disorders of ear	UKBB	European	0.002	4.50×10^{-6}
rs73493965	T	C	Other disorders of ear	UKBB	European	0.002	4.52×10^{-6}
rs112039104	A	C	Other disorders of ear	UKBB	European	0.002	4.72×10^{-6}
rs113398041	T	C	Other disorders of ear	UKBB	European	−0.002	4.73×10^{-6}
rs73493975	A	G	Other disorders of ear	UKBB	European	−0.002	4.95×10^{-6}
rs61058460	T	C	Other disorders of ear	UKBB	European	−0.002	5.32×10^{-6}
rs61468896	T	C	Other disorders of ear	UKBB	European	0.002	5.37×10^{-6}
rs77982873	T	C	Other disorders of ear	UKBB	European	−0.002	5.39×10^{-6}
rs77377087	A	T	Other disorders of ear	UKBB	European	−0.002	5.39×10^{-6}
rs11465408	A	G	Cause of death: inquest adjourned death	UKBB	European	0.007	5.44×10^{-6}
rs17159887	T	C	Other disorders of ear	UKBB	European	−0.002	5.89×10^{-6}
rs57889796	T	C	Other disorders of ear	UKBB	European	−0.002	5.99×10^{-6}
rs73493971	A	G	Cause of death: bladder, unspecified	UKBB	European	−0.084	6.11×10^{-6}
rs111716431	A	G	Cause of death: cerebrum, except lobes and ventricles	UKBB	European	0.017	6.11×10^{-6}
rs11465411	A	G	Other surgical follow-up care	UKBB	European	−0.001	6.20×10^{-6}
rs57668221	C	G	Other disorders of ear	UKBB	European	−0.002	6.21×10^{-6}
rs11465410	T	C	Other surgical follow-up care	UKBB	European	−0.001	6.49×10^{-6}
rs190601505	A	G	Cause of death: bladder, unspecified	UKBB	European	0.082	7.70×10^{-6}
rs11465395	C	G	Self-reported parathyroid hyperplasia or adenoma	UKBB	European	0.002	7.77×10^{-6}
rs111716431	A	G	Other disorders of ear	UKBB	European	0.002	7.84×10^{-6}
rs73493973	A	G	Self-reported parathyroid hyperplasia or adenoma	UKBB	European	0.002	8.78×10^{-6}
rs73493968	T	C	Self-reported parathyroid hyperplasia or adenoma	UKBB	European	−0.002	8.97×10^{-6}
rs71534255	A	C	Other acquired deformities of musculoskeletal system and connective tissue	UKBB	European	0.004	9.36×10^{-6}
rs73493966	C	G	Self-reported parathyroid hyperplasia or adenoma	UKBB	European	−0.002	9.46×10^{-6}
rs536227604	T	C	Cause of death: vascular disorder of intestine, unspecified	UKBB	European	−0.065	9.70×10^{-6}
rs112039104	A	C	Self-reported parathyroid hyperplasia or adenoma	UKBB	European	0.002	9.77×10^{-6}
rs113398041	T	C	Self-reported parathyroid hyperplasia or adenoma	UKBB	European	−0.002	9.80×10^{-6}
rs113836492	A	G	Cause of death: thoracic aortic aneurysm, ruptured	UKBB	European	0.011	9.81×10^{-6}
rs73493975	A	G	Self-reported parathyroid hyperplasia or adenoma	UKBB	European	−0.002	9.97×10^{-6}

Table S3. The top 100 CD209 expression associated traits identified by PheWAS in lung tissues.

Phenotype	phenotype_source	zscore	effect_size	pvalue
Neutrophill percentage	UK Biobank	−4.159	−0.604	3.20×10^{-5}
Neutrophill count	UK Biobank	−4.108	−0.100	4.00×10^{-5}
Diagnoses - main ICD10: L92 Granulomatous disorders of skin and subcutaneous tissue	UK Biobank	−3.840	−0.002	1.23×10^{-4}
Granulomatous disorders of skin and subcutaneous tissue	UK Biobank	−3.840	−0.002	1.23×10^{-4}
Granulocyte Count	Astle_et_al_2016	−3.822		1.32×10^{-4}
Manic/hyper symptoms: I was more creative or had more ideas than usual	UK Biobank	−3.819	−0.081	1.34×10^{-4}
Sum Neutrophil Eosinophil Count	Astle_et_al_2016	−3.745		1.80×10^{-4}
Lymphocyte percentage	UK Biobank	3.617	0.455	2.98×10^{-4}
Sum Basophil Neutrophil Count	Astle_et_al_2016	−3.556		3.77×10^{-4}
Myeloid White Cell Count	Astle_et_al_2016	−3.534		4.09×10^{-4}
Neutrophil Count	Astle_et_al_2016	−3.468		5.24×10^{-4}
Duration of fitness test	UK Biobank	3.400	0.102	6.74×10^{-4}
Diagnoses - main ICD10: Z45 Adjustment and management of implanted device	UK Biobank	3.391	0.005	6.97×10^{-4}
Impact on normal roles during worst period of anxiety	UK Biobank	−3.390	−0.169	6.99×10^{-4}
White Blood Cell Count	Astle_et_al_2016	−3.342		8.32×10^{-4}
Monocyte percentage	UK Biobank	3.231	0.149	1.24×10^{-3}
Long-standing illness, disability or infirmity	UK Biobank	−3.194	−0.026	1.40×10^{-3}
Recent feelings of tiredness or low energy	UK Biobank	−3.191	−0.077	1.42×10^{-3}
Treatment/medication code: amoxicillin (20003_1141180226)	UK Biobank	−3.148	−0.002	1.64×10^{-3}
Thoughts of death during worst depression	UK Biobank	−3.138	−0.064	1.70×10^{-3}
Non-cancer illness code, self-reported: pleurisy	UK Biobank	−3.109	−0.003	1.88×10^{-3}
Other disorders involving the immune mechanism, not elsewhere classified	UK Biobank	−3.020	−0.001	2.53×10^{-3}
Stenosis and insufficiency of lacrimal passages	UK Biobank	2.995	0.002	2.75×10^{-3}
White blood cell (leukocyte) count	UK Biobank	−2.968	−0.105	3.00×10^{-3}
Diagnoses - main ICD10: D89 Other disorders involving the immune mechanism, not elsewhere classified	UK Biobank	−2.964	−0.001	3.03×10^{-3}
Diagnoses - main ICD10: D09 Carcinoma in situ of other and unspecified sites	UK Biobank	−2.960	−0.001	3.08×10^{-3}
Treatment/medication code: diprosalic ointment (20003_1140882618)	UK Biobank	2.944	0.001	3.24×10^{-3}
Never eat eggs, dairy, wheat, sugar: Wheat products	UK Biobank	−2.913	−0.008	3.58×10^{-3}
Hospital episode type: General episode	UK Biobank	−2.912	−0.022	3.60×10^{-3}
Spondylolisthesis/Spondylolysis	UK Biobank	−2.887	−0.002	3.89×10^{-3}
Diagnoses - main ICD10: C49 Malignant neoplasm of other connective and soft tissue	UK Biobank	−2.867	−0.001	4.15×10^{-3}
Diagnoses - main ICD10: M43 Other deforming dorsopathies	UK Biobank	−2.864	−0.002	4.18×10^{-3}
Job coding: educational assistant, classroom assistant, special needs helper (22601_61243409)	UK Biobank	−2.859	−0.015	4.25×10^{-3}
Job coding: credit controller/supervisor (22601_41213252)	UK Biobank	2.838	0.005	4.54×10^{-3}
Length of time at current address	UK Biobank	2.824	0.530	4.74×10^{-3}

Diagnoses - main ICD10: S43 Dislocation, sprain and strain of joints and ligaments of shoulder girdle	UK Biobank	−2.795	−0.001	5.19×10^{-3}
Treatment/medication code: vitamin b6 preparation (20003_1140909874)	UK Biobank	−2.788	−0.002	5.31×10^{-3}
Destinations on discharge from hospital (recoded): Usual Place of residence	UK Biobank	−2.787	−0.023	5.32×10^{-3}
Types of transport used (excluding work): Walk	UK Biobank	−2.778	−0.024	5.47×10^{-3}
Leg fat percentage (right)	UK Biobank	2.775	0.262	5.53×10^{-3}
Traumatic subarachnoid haemorrhage	UK Biobank	−2.774	−0.001	5.53×10^{-3}
Non-cancer illness code, self-reported: osteopenia	UK Biobank	−2.760	−0.002	5.78×10^{-3}
Astigmatism angle (left)	UK Biobank	−2.755	-5.147	5.87×10^{-3}
Treatment/medication code: frusemide (20003_1140866116)	UK Biobank	−2.729	−0.002	6.34×10^{-3}
ECG, load	UK Biobank	2.725	1.935	6.43×10^{-3}
Sum Eosinophil Basophil Count	Astle_et_al_2016	−2.706		6.81×10^{-3}
#Deforming dorsopathies	UK Biobank	−2.674	−0.002	7.50×10^{-3}
Loud music exposure frequency	UK Biobank	−2.668	−0.057	7.63×10^{-3}
Treatment/medication code: persantin 25mg tablet (20003_1140861780)	UK Biobank	2.667	0.001	7.65×10^{-3}
ECG, phase time	UK Biobank	2.656	5.040	7.92×10^{-3}
Treatment/medication code: vesicare 5mg tablet (20003_1141200384)	UK Biobank	2.649	0.001	8.08×10^{-3}
Job SOC coding: Vehicle body builders and repairers	UK Biobank	−2.648	−0.004	8.11×10^{-3}
Difficulty concentrating during worst depression	UK Biobank	−2.645	−0.046	8.18×10^{-3}
Other and unspecified granulomatous disorders of skin and subcutaneous tissue	UK Biobank	−2.639	−0.001	8.32×10^{-3}
Job coding: civil service executive officer, revenue/tax executive or officer, fraud inspector or officer, job centre adviser (22601_41113204)	UK Biobank	−2.632	−0.017	8.49×10^{-3}
Treatment/medication code: vascalpha 5mg m/r tablet (20003_1141190160)	UK Biobank	2.616	0.001	8.89×10^{-3}
Job SOC coding: Civil Service executive officers	UK Biobank	−2.611	−0.017	9.04×10^{-3}
Mouth/teeth dental problems: Mouth ulcers	UK Biobank	−2.603	−0.014	9.24×10^{-3}
Diagnoses - main ICD10: E86 Volume depletion	UK Biobank	2.599	0.001	9.35×10^{-3}
Diseases of veins, lymphatic vessels and lymph nodes, not elsewhere classified	UK Biobank	2.594	0.008	9.48×10^{-3}
Non-cancer illness code, self-reported: insomnia	UK Biobank	−2.568	−0.001	1.02×10^{-2}
Activities undertaken to treat depression: Talking therapies, such as psychotherapy, counselling, group therapy or CBT	UK Biobank	−2.567	−0.031	1.03×10^{-2}
Job SOC coding: Educational assistants	UK Biobank	−2.550	−0.014	1.08×10^{-2}
Other and unspecified disorders of skin and subcutaneous tissue	UK Biobank	−2.545	−0.004	1.09×10^{-2}
Multiple worries during worst period of anxiety	UK Biobank	−2.528	−0.059	1.15×10^{-2}
Age at death	UK Biobank	2.526	0.516	1.15×10^{-2}
Sensitivity / hurt feelings	UK Biobank	−2.522	−0.021	1.17×10^{-2}
Time spent doing vigorous physical activity	UK Biobank	−2.521	−0.159	1.17×10^{-2}
Seen doctor (GP) for nerves, anxiety, tension or depression	UK Biobank	−2.520	−0.020	1.17×10^{-2}
Diagnoses - main ICD10: N90 Other noninflammatory disorders of vulva and perineum	UK Biobank	−2.520	−0.002	1.17×10^{-2}

Non-cancer illness code, self-reported: labyrinthitis	UK Biobank	−2.515	−0.001	1.19×10^{-2}
Job SOC coding: Upholsterers	UK Biobank	2.512	0.003	1.20×10^{-2}
Maximum workload during fitness test	UK Biobank	2.503	3.244	1.23×10^{-2}
Diagnoses - main ICD10: N32 Other disorders of bladder	UK Biobank	−2.495	−0.005	1.26×10^{-2}
Number of trend entries	UK Biobank	2.491	2.562	1.27×10^{-2}
Job SOC coding: Textile process operatives	UK Biobank	−2.483	−0.005	1.30×10^{-2}
Leg fat percentage (left)	UK Biobank	2.481	0.223	1.31×10^{-2}
Diseases of the musculoskeletal system and connective tissue	UK Biobank	−2.480	−0.017	1.31×10^{-2}
Fibromyalgia related co-morbidities	UK Biobank	−2.472	−0.003	1.34×10^{-2}
Treatment/medication code: omacor 1g capsule (20003_1141181868)	UK Biobank	−2.469	−0.002	1.35×10^{-2}
Depression possibly related to stressful or traumatic event	UK Biobank	−2.455	−0.043	1.41×10^{-2}
Treatment/medication code: salbutamol 100micrograms spacehaler (20003_1140926606)	UK Biobank	2.451	0.003	1.43×10^{-2}
Eosinophil Count	Astle_et_al_2016	−2.444		1.45×10^{-2}
Hypergammalglobulinemia, unspecified	UK Biobank	−2.444	−0.001	1.45×10^{-2}
Diagnoses - main ICD10: K11 Diseases of salivary glands	UK Biobank	−2.435	−0.002	1.49×10^{-2}
Other disorders of skin and subcutaneous tissue, not elsewhere classified	UK Biobank	−2.420	−0.004	1.55×10^{-2}
Diagnoses - main ICD10: L98 Other disorders of skin and subcutaneous tissue, not elsewhere classified	UK Biobank	−2.420	−0.004	1.55×10^{-2}
Ankle spacing width	UK Biobank	2.418	0.216	1.56×10^{-2}
Diagnoses - main ICD10: C67 Malignant neoplasm of bladder	UK Biobank	−2.410	−0.003	1.60×10^{-2}
Job coding: taxi or cab driver, chauffeur, hearse driver (22601_82142611)	UK Biobank	2.409	0.004	1.60×10^{-2}
Illnesses of father: Stroke	UK Biobank	2.406	0.016	1.62×10^{-2}
Basophill count	UK Biobank	−2.405	−0.035	1.62×10^{-2}
Job coding: textile machine operator, assembler, spinner, foreman (22601_81132710)	UK Biobank	−2.399	−0.004	1.65×10^{-2}
Job SOC coding: Taxi, cab drivers and chauffeurs	UK Biobank	2.397	0.004	1.65×10^{-2}
Diagnoses - main ICD10: J38 Diseases of vocal cords and larynx, not elsewhere classified	UK Biobank	−2.395	−0.002	1.66×10^{-2}
Job coding: upholsterer, coach trimmer, curtain maker, mattress maker (22601_54122878)	UK Biobank	2.387	0.003	1.70×10^{-2}
Other serious medical condition/disability diagnosed by doctor	UK Biobank	−2.385	−0.017	1.71×10^{-2}
Used an inhaler for chest within last hour	UK Biobank	−2.376	−0.004	1.75×10^{-2}
Treatment/medication code: kapake tablet (20003_1140864070)	UK Biobank	2.367	0.001	1.79×10^{-2}
Birth Weight	EGG	−2.366		1.80×10^{-2}

Table S4. Mendelian Randomization inferred causal effect of plasma expression of DC-SIGN on cell count and percentage.

Outcome	nsnp	b	se	pval
White blood cell (leukocyte) count ǀǀ id:ukb-d-30000_irnt	3	−0.031	0.003	5.40×10^{-27}
Neutrophill count ǀǀ id:ukb-d-30140_irnt	3	−0.027	0.003	1.38×10^{-16}
Red blood cell count ǀǀ id:ieu-a-275	3	−0.014	0.003	2.31×10^{-7}
Lymphocyte percentage ǀǀ id:ukb-d-30180_irnt	3	0.017	0.004	5.29×10^{-5}
Granulocyte count ǀǀ id:ebi-a-GCST004614	2	−0.042	0.011	1.01×10^{-4}
Sum neutrophil eosinophil counts ǀǀ id:ebi-a-GCST004613	2	−0.041	0.011	1.41×10^{-4}
Myeloid white cell count ǀǀ id:ebi-a-GCST004626	2	−0.040	0.011	2.61×10^{-4}
Sum basophil neutrophil counts ǀǀ id:ebi-a-GCST004620	2	−0.039	0.011	3.27×10^{-4}
Granulocyte percentage of myeloid white cells ǀǀ id:ebi-a-GCST004608	2	−0.039	0.011	3.63×10^{-4}
Neutrophil count ǀǀ id:ebi-a-GCST004629	2	−0.038	0.011	4.83×10^{-4}
White blood cell count ǀǀ id:ebi-a-GCST004610	2	−0.037	0.01	6.52×10^{-4}
Lymphocyte count ǀǀ id:ukb-d-30120_irnt	3	−0.011	0.003	1.69×10^{-3}
Red blood cell (erythrocyte) count ǀǀ id:ukb-d-30010_irnt	3	−0.030	0.010	1.94×10^{-3}
Monocyte percentage of white cells ǀǀ id:ebi-a-GCST004609	2	0.031	0.012	8.52×10^{-3}
Eosinophil counts ǀǀ id:ebi-a-GCST004606	2	−0.028	0.011	9.18×10^{-3}
Monocyte count ǀǀ id:ukb-d-30130_irnt	3	−0.042	0.017	1.12×10^{-2}
Neutrophil percentage of white cells ǀǀ id:ebi-a-GCST004633	2	−0.025	0.011	2.28×10^{-2}
Eosinophill percentage ǀǀ id:ukb-d-30210_irnt	3	−0.010	0.004	2.47×10^{-2}
Lymphocyte percentage of white cells ǀǀ id:ebi-a-GCST004632	2	0.023	0.011	3.48×10^{-2}

nsnp: number of SNPs used in analysis; b: beta coefficient; se: standard error
Inverse variance weighted method was used in this analysis.
